# Primary care follow-up of patients after attending a fracture liaison service: an integrative review

**DOI:** 10.1007/s11657-025-01521-8

**Published:** 2025-05-13

**Authors:** Michael J. Bennett, Jacqueline R. Center, Lin Perry

**Affiliations:** 1 Department of Endocrinology, Prince of Wales Hospital, Randwick, NSW Australia; 2 Department of Endocrinology, St Vincent’s Hospital, Darlinghurst, NSW Australia; 3 Randwick Clinical Campus, UNSW Medicine, Sydney, NSW Australia; 4 The Garvan Institute of Medical Research, Darlinghurst, NSW Australia; 5 St Vincent’s Healthcare Clinical School, School of Clinical Medicine, UNSW, Sydney, NSW Australia; 6 Faculty of Health, University of Technology Sydney, Ultimo, NSW Australia; 7 Prince of Wales Hospital & Community Health Services, Randwick, NSW Australia

**Keywords:** Fracture liaison services, Primary care, Osteoporosis, Healthcare integration, Healthcare policy

## Abstract

**Summary:**

Based on a small number of predominantly low-to-moderate quality studies with moderate-to-high risk of bias, the FLS-to-primary care transition is portrayed as a challenging time for patients, GPs, and FLS clinicians, who experience numerous barriers to care continuation and coordination at this care juncture.

**Purpose:**

Continuity and coordination of care between fracture liaison services (FLS) and primary care is required for optimal long-term osteoporosis care. This study aims to explore (1) how patients and healthcare providers (HCPs) experience the FLS to primary care transition, (2) the barriers and facilitators to primary care follow-up after FLS, and (3) interventions that enhance integration of FLS with primary care.

**Methods:**

An integrative review was performed and reported in accordance with the Preferred Reporting Items for Systematic Review and Meta Analysis (PRISMA) Statement 2020. Online bibliographic databases were searched using the terms “osteoporosis”, “primary care”, and “fracture liaison services” and related terms for original English-language studies conducted between January 1, 2003 and December 29, 2023. Manuscripts were assessed for relevance using pre-defined criteria, and for quality and bias using validated instruments. Thematic analysis was used to extract key themes relevant to each research question.

**Results:**

Overall, 14 relevant manuscripts were identified. Among the four studies that addressed patient and HCP experience of the FLS-to-primary care transition, five key themes emerged: (1) time and workload pressures, (2) limited confidence in primary care follow-up, (3) GP knowledge gaps, (4) siloed or disconnected care, and (5) communication issues. Twelve studies addressed barriers and facilitators to primary care follow-up after FLS, which fell into five themes: (1) patient knowledge and understanding (2) miscommunication and misinformation, (3) understanding roles and responsibilities, (4) GP engagement, and (5) GP-patient relationship. Additionally, single studies suggested healthcare policies and funding, accessing primary care from residential facilities, and GP gender influenced primary care follow-up. Five studies detailed interventions to enhance FLS-to-primary care integration. GP education and patient reminders, delivered as part of a multifaceted intervention, appeared to improve integration of acute and primary post-fracture care; however, the contribution of these individual interventions to outcomes remains unclear. While telephone coaching and bone marker monitoring were identified as potential interventions, there was insufficient evidence to conclude they are effective.

**Conclusion:**

Available evidence was generally low-to-moderate quality with moderate-to-high risk of bias. Integration of the available evidence portrays the FLS-to-primary care transition as a challenging time for patients, GPs, and FLS clinicians, who experience a multitude of barriers to care continuation and coordination. There is insufficient data to support any single intervention as effective for enhancing care coordination beyond those considered standard components of FLS models. Knowledge gaps exist regarding the patient experience of the FLS-to-primary care transition, facilitators to primary care follow-up, interventions to support FLS integration with primary care, and how such information may be integrated to optimise care for patients with osteoporosis.

**Supplementary Information:**

The online version contains supplementary material available at 10.1007/s11657-025-01521-8.

## Introduction

Osteoporosis is a chronic skeletal disease characterised by low bone mass and microarchitectural deterioration resulting in bone fragility and increased fracture risk [1]. It is thought to affect 23.1% of women and 11.7% of men globally [2] with an estimated one in three women and one in five men over 50 years of age sustaining an osteoporosis-related fracture [3].

Osteoporosis-related fractures, also termed *fragility fractures*, are a leading cause of morbidity, mortality, and healthcare expenditure. Mortality risk increases in the 12 months after an osteoporotic fracture and remains elevated for five or more years [4] with the relative risk of death being greatest for fractures of the hip (2.1 for women and 2.9 for men) and vertebrae (2.0 for women and 2.5 for men) [5]. Those who do not die following a fracture may experience chronic pain, disability, or loss of independence necessitating institutionalisation [6]. In 2016 in the EU6 (France, Germany, Italy, Spain, UK, and Sweden), fragility fractures were responsible for 5.5 years of life lost (YLL) and 15.1 years of life lost due to disability (YLD) per 1000 people [7]. The economic costs of fractures is similarly staggering; in 2017, fragility fractures cost €37.5 billion in the EU6 and US$2.44 billion in Australia [7, 8]; in the USA, mean healthcare costs in the year after a fragility fracture exceed US$30,000 per person (2017) [9]. Much of the human and economic burden of osteoporosis is preventable through timely diagnosis and treatment.

Following a fragility fracture, the risk of subsequent fracture rises steeply, and a vicious cycle of recurrent fractures can be established [10]. A fragility fracture therefore provides an opportunity for medical intervention to break the fracture cycle. Despite the widespread availability of safe and effective osteoporosis treatments [11], the majority of those with a fragility fracture go undiagnosed and untreated, a phenomenon termed the *osteoporosis treatment gap* [12]. This enduring gap has complex and multifactorial origins [13] and in some regions has actually widened in recent years [7, 14]. The net effect is a largely preventable burden of re-fractures leading to rising human and economic costs. In New South Wales, for example, the incidence of re-fractures increased from 8774 in 2008 to 14,323 in 2018, costing AU$130 million and AU$194 million each year, respectively [15].

Fracture liaison services (FLS) are models of care for secondary fracture prevention that have been developed to address the global osteoporosis treatment gap. FLS take a systematic approach to *identify* patients following a fragility fracture, *inform* (educate) them of their fracture risk, deliver bone health *education*, coordinate *investigations* into their bone health, and *initiate* timely, evidence-based, fracture risk-reducing treatments. Several FLS models exist, which vary in service intensity [16]. Type A models (gold standard) identify, inform, investigate, and initiate treatment. Type B models identify, inform, and investigate but delegate treatment initiation to primary care. Type C models identify and inform patients before notifying their general practitioner of the recommendation for further assessment. Type D models perform identification and information functions only. Compared to usual care, FLS have been shown to improve osteoporosis testing and treatment rates and reduce re-fractures and mortality in a cost-effective manner [17–19]. It has been reported that at least 19,000 fractures could be prevented each year in the EU6 if all patients over 50 years of age with a fragility fracture were enrolled in an FLS [7]. However, attendance needs to be followed by treatment initiation, and FLS treatment initiation rates, while substantially higher than usual care, are suboptimal and range from 46% for type A models, to 8% for type D models [13]. Poor treatment uptake and treatment discontinuation remain challenges that threaten service effectiveness.

Osteoporosis requires a lifelong approach to monitoring and management. Current treatments are non-curative and their antifracture effects wane following discontinuation. Medication persistence is therefore likely to be a key determinant of the long-term success of FLS. While many FLS have reported short-term persistence rates between 66.4 to 88% [20–23], fewer studies have examined longer term medication persistence, which appears to decline steadily over time, with only 45% of patients continuing treatment to 5 years [24, 25].

Hence, FLS are chiefly concerned with short-term case identification and treatment initiation and rely on primary care to deliver long-term osteoporosis care. In recent years, there have been growing calls for health systems to adopt a more integrated approach to bone health management [26, 27] and clinical standards for FLS in several countries now recognise the need to deliver services as part of a broader, multidisciplinary, integrated care pathway [28–30]. Despite this, little is known about the nature of the FLS-to-primary care transition and the experiences of patients and healthcare providers at this junction in care. Moreover, primary care attendance is an infrequently reported FLS performance indicator, with only a few small studies providing an incomplete view. Laslett et al. reported 57–71% of patients consulted their general practitioner (GP) following fracture; however, the reasons for doing so were not reported [31]. Bliuc et al. reported 20% of patients enrolled in a type B/C FLS attended their GP for osteoporosis-related follow-up within 3 months of fracture [32]. Blonk et al. reported 82% of patients attended their GP 3 months after attending a type B FLS [33]. These studies provide an indication of the widely varying but generally inadequate proportion of patients who attend for at least an initial GP consultation for post-fracture care. They reveal an absence of data on the care continuity that is required for effective treatment of osteoporosis.

This review aimed to address this important gap by answering the following research questions:

1. How do patients, and the GPs and FLS clinicians who provide care for these patients, experience the transition of patient care from hospital-based FLS service to primary care?
2. What are the barriers and facilitators to primary care follow-up for patients who have attended an FLS service?
3. What interventions have been applied with the aim of enhancing integration (or care coordination) of hospital-based secondary fracture prevention services with primary care?

## Methods

An integrative review was chosen to enable integration differing types of data from multiple research designs [34]. The review was reported in accordance with the Preferred Reporting Items for Systematic Review and Meta Analysis (PRISMA) Statement 2020 [35].

### Information sources

An electronic search for relevant studies was performed on December 30, 2023 using the following databases: Embase (Ovid), PubMed, Cumulative Index to Nursing and Allied Health Literature (CINAHL) (Ebsco*Host*), Social Sciences Citation Index (SSCI) (Web of Science), and Applied Social Sciences Index and Abstracts (ASSIA) (ProQuest). A “snowball search” of reference lists from eligible full-text articles was also conducted along with a search for “similar articles” using Google Scholar.

### Search strategy

English language original research articles, including human participants aged ≥ 45 years, published from January 1, 2003 to December 29, 2023 were included. As the first report [36] of an FLS was published in 2003, this date was chosen as the inception date of FLS, and studies prior to this date were excluded. Both qualitative and quantitative studies were included. Database searches were performed using all fields (including title, abstract, and key words) and included the following search terms: “osteoporosis” (or “fragility fracture” or “minimal trauma fracture” or “spine fracture” or “hip fracture”) and “primary care” (or “general practice” or “general practitioner” or “primary care provider” or “primary care physician”) and “fracture liaison” (or “refracture prevention” or “secondary fracture prevention” or “osteoporosis clinic” or “bone clinic”), including related terms. For detailed search terms and applied filters see Supplement 1.

### Eligibility criteria

To be eligible for inclusion, studies must have included a population and outcome of interest. For research question 3 (RQ3), studies must also have included an intervention of interest.

- Population:
  i. Adult patients (> 45 years of age) who undergo investigation for osteoporosis following a hospital presentation with a fragility fracture
  ii. Primary care physicians (or GPs) who care for these patients
  iii. Clinicians working in hospital-based (inpatient or outpatient) fracture liaison services (FLS) who care for these patients
- Outcome: the transition of care from hospital-based secondary fracture prevention services to primary care. Research question 1 (RQ1) sought participants’ experience of this; research question 2 (RQ2) sought factors that barred or facilitated this; RQ3 sought interventions that enhanced this. Studies could include an FLS of any subtype (A to D) [16].
  i. For RQ1 and RQ2, the transition was required to be acknowledged but not required to be formally assessed or measured.
  ii. RQ3 only sought an intervention of interest in the form of any activity designed to enhance this transition in care.
- Intervention (applicable to RQ3 only): any intervention designed or delivered with the intention of enhancing the integration of care between FLS and primary care.

### Exclusion criteria

Publications in the form of conference abstracts, letters to the editor, opinion articles, study protocols, and clinical guidelines were excluded. Similarly, articles that lacked sufficient methodological details to assess the population, intervention, or outcome of interest with regard to the research questions were excluded. Studies evaluating interventions considered standard elements of any FLS (e.g. a letter sent to the patient’s GP summarising FLS recommendations, patient invitation to participate in FLS, supported access to investigations, routine telephone calls from FLS staff to assess adherence) or comparing one model of FLS against another using recognised key performance indicators were excluded. Studies evaluating the effect of population-level interventions, such as public health campaigns, were also excluded.

*FLS* were defined as any secondary fracture prevention services that included a dedicated coordinator and provided all of the following: (a) active case finding of patients who have presented to hospital following a fragility fracture; (b) investigation of bone health (such as dual x-ray absorptiometry (DXA), skeletal x-ray, pathology investigations), which may be in the form of a referral for investigation or advice to the patient (or their GP) to arrange investigation; (c) provision of information to the patient concerning osteoporosis or fracture risk; and (d) initiation of specific evidence-based anti-osteoporosis treatment(s), which may be in the form of a prescription or advice to the patient (or their GP) to initiate treatment.

*Integration* was defined as any process, method, or model designed to improve patient care and experience through improved coordination between acute and primary care [37].

### Data collection and selection process

Citations were imported into EndNote X9.3.3 and duplicates and conference abstracts were removed. Titles and abstracts were manually screened according to the above criteria. A second researcher independently reviewed 10% of the abstracts and cases of discordance were resolved through discussion. Full-text versions of all eligible manuscripts were reviewed by two independent reviewers (MB, LP), with discordance resolved through discussion. Excluded manuscripts were recorded along with the reasons for exclusion. Author names, study locations, and dates were examined to identify any manuscripts reporting information from the same study. In these instances, only the most relevant study was included, as determined through discussion by both reviewers.

A single reviewer (MB) performed data extraction using a data extraction table, which was developed and refined throughout the review process. A second reviewer (LP) verified the recorded data for accuracy. The following information was collected from included studies: (a) author, year of publication, and country of study origin; (b) number of participants and their characteristics; (c) type of FLS (A to D) employed; (d) study aims and design; (e) outcomes; and (f) details of any study intervention. For qualitative studies, all relevant participant quotes and themes were extracted.

### Study risk of bias (RoB) assessment and reporting

Each included study was assessed independently for bias by two reviewers (MB, LP) applying the mixed-methods appraisal tool (MMAT) [38]. An overall methodological rating of 0, 25, 50, 75, and 100 (where 100 indicates the highest and 0 indicates the lowest quality) was assigned to each study based on the results of the MMAT evaluation (Table 1 and Supplement 2). No papers were excluded following RoB assessment.

**Table 1:** Characteristics of included studies

Author (year)	Design	Sample size	Country	Population	FLS type	Aims and details of intervention	RoB score^1^	Quality score^2^
Bennett MJ, Center JR, Perry L (2023) [40]	Qualitative study	25 participants	Australia	7 FLS clinicians 11 GPs 7 Patients	A	Map service processes and factors influencing integration of post-clinic care, identifying barriers, supports, and opportunities for seamless health care	100	Moderate
Bishop S, Narayanasamy MJ, Paskins Z et al. (2023) [41]	Qualitative study	23 participants	United Kingdom	9 GPs 11 FLS (or bone clinic) clinicians, including 8 medical specialists and 3 nurses 3 nurses working in a community infusion service	Not reported	Investigate clinicians’ experiences prescribing different forms of bisphosphonate medication, focusing on how treatment decisions are made in practice and how this relates to ongoing processes of treatment and care	100	High
Bliuc D, Eisman J, Center JR (2006) [32]	Quantitative randomised trial	159 participants	Australia	79 patients (intervention 1) 80 patients (intervention 2)	B/C	Determine whether an information-based intervention could change post-fracture management of osteoporosis Secondary aim: define participant- and doctor-related barriers to osteoporosis management Intervention 1: personalised letter detailing participant’s risk factors for osteoporosis and recommended follow-up with their primary care physician Intervention 2: the same personalised letter but included an offer of a free BMD assessment	50	High
Blonk M, Erdtsieck RJ, Wernekinck MGA et al. (2007) [33]	Quantitative descriptive study	1058 participants	Netherlands	804 women 254 men	B	Investigate the 1-year efficacy of a fracture and osteoporosis clinic in terms of early diagnosis and treatment of osteoporosis and 3-month compliance with treatment advice	50	Moderate
Casado E, Blanch J, Carbonell C et al. (2021) [42]	Qualitative (Delphi) study	75 experts	Spain	Experts from various specialties: 18 Rheumatology 16 Primary care 11 Geriatrics 6 Internal medicine	Not reported	Reach a consensus among multidisciplinary experts on the best measures required to optimise fragility fracture management of patients in Spain	100	Moderate
Cranney A, Lam M, Ruhland L et al. (2008) [45]	Quantitative randomised trial	270 participants	Canada	125 women (intervention) 145 women (control)	C	Evaluate a multifaceted intervention designed to improve management of osteoporosis in older women with recent wrist fractures Intervention: personalised information targeted PCP and patient, mailed to both 2 weeks and 2 months post fracture. The PCP letter notified their patient’s recent wrist fracture, highlighted possible links to osteoporosis and recommended assessment for this. A two-page algorithm and educational tool summarised treatment options, benefits and risks. The patient letter recommended scheduling a follow-up visit with their PCP to discuss osteoporosis and included a 1-page checklist of risks for fractures to calculate 5-year fracture risk Comparison: usual care	75	High
Drew S, Judge A, Cooper C et al. (2016) [43]	Qualitative study	43 participants	United Kingdom	8 Fracture prevention nurses 4 Ortho-geriatricians 4 Geriatricians 2 GP osteoporosis specialists 5 trauma Orthopaedic surgeons 2 Orthopaedic nurses 2 Matrons 8 Rheumatologists 1 Falls coordinator 1 Falls nurse 1 BMD specialist 5 Service managers	Not reported	Identify the elements of care of hip fracture patients that health professionals think are most effective in preventing secondary fractures after hip fracture	100	Moderate
Inderjeeth CA, Glennon DA, Poland KE et al. (2010) [48]	Quantitative descriptive study	652 participants	Australia	306 GPs 200 patients 103 Physicians 43 Surgeons	C/?^3^	Implement and evaluate a multimodal intervention to improve osteoporosis treatment in patients with a fragility fracture Intervention: implementing guideline-based care only, so not eligible in relation to RQ3	25	Low
Jaglal SB, Cameron C, Hawker GA et al. (2006) [49]	Mixed-methods study	259 participants	Canada	Focus groups with 21 women (patients) 26 family physicians 34 key informants Telephone survey of 178 hospital nurse managers (or equivalent)	Not reported	Develop an integrated care model for patients who have had a low-trauma fracture and to identify barriers and opportunities for post-fracture care interventions	100	Moderate
Laslett LL, Whitham JM, Gibb C et al. (2007) [31]	Quantitative non-randomised study	56 participants	Australia	28 participants in pre-intervention group 28 participants in usual care group	Not classifiable^4^	Compare patient outcomes before and after implementation of a clinical protocol for low-trauma fractures Intervention: patients received individual counselling by a hospital pharmacist and were provided with an “osteoporosis pack” containing educational material and letter to their GP. Pharmacist recommended prescription of osteoporosis medications (calcium, vitamin D, and bisphosphonate) by junior doctors during patient admission	50	Low
Luc M, Corriveau H, Boire G et al. (2018) [50]	Mixed-methods study	354 participants	Canada	306 women 48 men	A	Identify factors associated with patients’ adherence at 12 months to various FLS recommendations including osteoporosis medication and bone health behaviours	75	Moderate
Sale JEM, Bogoch E, Hawker G et al. (2014) [44]	Qualitative study	25 patients	Canada	22 women 3 men	C	Examine patients’ experiences and actions regarding osteoporosis investigation and treatment after they were screened through an FLS; explore potential barriers to post-fracture secondary prevention experienced by, or influencing, patients before and after fracture risk assessment	100	High
Vaculik J, Stepan JJ, Dungl P et al. (2017) [47]	Quantitative non-randomised study	207 participants	Czech Republic	111 patients (intervention 1) 96 patients (intervention 2)	C	Assess whether an individual rather than a general recommendation on osteoporosis treatment addressed to a hip fracture patient’s GP would lead to better osteoporosis management Intervention 1: general recommendations on osteoporosis treatment and fracture prevention provided in a discharge report addressed to the GP Intervention 2: individually detailed set of recommendations on osteoporosis examination, treatment, and fracture prevention, which was also provided in the discharge report addressed to the GP	25	Low
Zinger G, Sylvetsky N, Levy Y et al. (2021) [46]	Quantitative randomised trial	200 participants	Israel	100 patients (intervention 1) 100 patients (intervention 2)	B/C	Test the effectiveness of an orthopaedic-driven intervention program in getting patients started on osteoporosis treatment Intervention 1: patients received printed information, a DXA scan, a specific treatment recommendation, and monthly phone calls for 4 months (equivalent to type B FLS plus phone coaching) Intervention 2: patients received a letter at the time of discharge encouraging their GP to start medication for osteoporosis (equivalent to type C FLS)	50	High

*FLS* fracture liaison service, *GP* general practitioner, *BMD* bone mineral density, *RoB* risk of bias

1. RoB assessment performed using the mixed-methods appraisal tool (MMAT). An overall rating of 0, 25, 50, 75, 100 (where 100 indicates to the highest and 0 indicates the lowest quality) was assigned to each study based on the results of the MMAT evaluation (Supplement 2)

2. Quality assessment performed using the Joanna Briggs Institute (JBI) critical appraisal tools. An overall quality score was determined accordingly: low quality (< 50%), moderate quality (50–70%), high quality (> 70%). This score was then modified based on reviewer assessment and discussion (Supplement 3)

3. Fracture patients were offered review by their GP (type C model of care) or review in the hospital “fragile bone clinic”. The model of care employed by the fragile bone clinic (type A or B) is not reported in the manuscript

4. The FLS intervention involved a coordinator (pharmacist) and included identification, education, and treatment initiation. No investigations were performed. Therefore, the intervention could be considered either a type A FLS (lacking an investigation component) or Type C FLS (with additional treatment initiation component)

### Synthesis methods

Given the nature of these research questions, and the predominance of qualitative and mixed-methods studies, a qualitative descriptive approach to analysis was employed. Inductive coding was used to analyse manuscripts for emergent themes, which were then mapped to each research question: experiences of the FLS-to-primary care transition (RQ1), barriers and facilitators to primary care follow-up (RQ2), and interventions to enhance integration (RQ3). For manuscripts that addressed more than one research question, data were independently analysed for each research question. A summary outcomes table was developed to present the findings of each study as they related to the research questions and a thematic analysis table to present the themes (and supporting quotes) relevant to each research question.

### Quality assessment

The Joanna Briggs Institute (JBI) Critical Appraisal Tools [39] were used to assess the methodological quality of each study. A total score for each study was calculated by dividing the number of affirmative responses by the number of relevant checklist items and multiplying by 100 to give a percentage. This initial score was used to group studies accordingly: low (< 50%), moderate (50–70%), and high (> 70%) quality. This score was then modified based on reviewer (MB, LP) assessment and discussion, resulting in an overall quality score (Table 1 and Supplement 3). No papers were excluded following quality assessment.

To maximise study rigour, techniques of reflexivity, data triangulation (to improve data conformability), and independent data verification by a co-researcher (to improve data dependability) were employed.

## Results

### Study selection

A search of five databases identified 1134 records. An additional 44 records were identified through citation searching, therefore a total of 1178 records were identified. Following removal of 216 duplicate records and 298 conference abstracts, 664 abstracts remained and of these 563 were not relevant (did not meet ≥ 1 study inclusion criteria or met ≥ 1 study exclusion criteria). Full-text manuscripts were assessed for the remaining 101 records, of which 87 were not relevant and one contained duplicate data (Supplement 4). Overall, 14 manuscripts were eligible; four addressed RQ1; 12 addressed RQ2; and five addressed RQ3.

### Study characteristics

Characteristics of included studies are shown in Table 1. The 14 analysed studies included five qualitative studies [40–44], seven quantitative studies (including three randomised trials [32, 45, 46], two non-randomised studies [31, 47], two descriptive studies [33, 48]), and two mixed-methods studies [49, 50]. Four studies were conducted in Australia [31, 32, 40, 48]; four in Canada [44, 45, 49, 50]; two in the UK [41, 43]; one each in the the Netherlands [33], Spain [42], the Czech Republic [47], and Israel [46]. Studies were conducted using a range of different FLS models; two type A models [40, 50], one type B model [33]; three type C models [44, 45, 47]; and three involved more than one model type [32, 46, 48]. In five studies, the type of FLS model was not reported or unclear [31, 41–43, 49]. Six studies included healthcare professionals (HCPs) as participants [40–43, 48, 49], all of which included GPs. The mean number of GP participants was 62 (SD 120.0). Eleven studies included patient participants (mean 232, SD 293.4).

Only seven studies [31, 32, 40, 44, 45, 47, 49] included a study aim that directly addressed one or more of this review’s research questions. The remaining seven studies included relevant data that were captured in a secondary or exploratory manner.

### Risk of bias in studies

Six studies had a RoB assessment score of 100 (indicating a low risk of study bias), two had a score of 75, and six had a score of 50 or less (moderate-to-high risk of study bias). There were concerns about the overall risk of study bias for two studies [47, 48]. The study by Inderjeeth et al. [48], which evaluated a multimodal intervention to improve osteoporosis treatment, did not report baseline participant characteristics, method of GP recruitment, or rationale for combining cohorts for their statistical analysis. Their study methods (in particular, patient-led treatment assignment) may have led to intrinsic differences in participant characteristics between groups, biasing study results. Similarly, the study by Vaculik et al. [47], which explored the effect of individualised vs general recommendations of osteoporosis treatment, relied on patient-recall of GP recommendations, which were not verified. Moreover, the study had a low response and high dropout rate and suffered from study group contamination as GPs may have provided care to patients from both study groups. Overall RoB scores for each study are listed in Table 1 and a summary of the individual components of the RoB assessment and comments for each study are shown in Supplement 2.

### Results of individual studies

Table 2 provides a text summary of findings for each study as they relate to the three research questions. Four studies addressed RQ1 [40, 41, 43, 48], 12 addressed RQ2 [31–33, 40–45, 48–50], and five addressed RQ3 [31, 41, 45–47].

**Table 2:** Study outcomes relating to each research question

Author (year)	RQ1—experience of the transition	RQ2—barriers and facilitators to GP follow-up	RQ3—interventions to enhance integration
Bennett MJ, Center JR, Perry L (2023) [40]	HCPs experienced frustration with delayed, absent, inaccessible, or otherwise poor-quality interprofessional communication between FLS and primary care A lack of suitable two-way communication between acute and primary care left FLS clinicians with no way of knowing whether their management plans were continued in primary care GPs had variable understanding of the role of the FLS and experienced role ambiguity when caring for patients transitioning to primary care Patients experienced specialist-led osteoporosis follow-up care as superior to GP-led care with regard to continuity, convenience, and quality of advice. They experienced GP-led osteoporosis care as “messy” (increased number of healthcare transitions), which may be particularly challenging for older persons	Barriers and facilitators to long-term post-fracture care: 1. Interprofessional communication 2. Understanding roles and responsibilities 3. GP-patient relationship 4. Patient knowledge, attitudes, and health behaviours 5. Patient education and treatment initiation 6. Healthcare policies, priorities and funding arrangements For HCPs, interprofessional communication issues and role ambiguity were principal barriers For patients, the absence of a strong GP–patient relationship, lack of perceived need to engage with a GP, and poor understanding of osteoporosis were key barriers	
Bishop S, Narayanasamy MJ, Paskins Z et al. (2023) [41]	GPs experienced uncertainty regarding treatment choices; they relied on the advice of osteoporosis specialists to inform their treatment decisions GPs experienced variation in the utility and accessibility of advice from secondary care specialists and some GPs were unsure how to act on advice In most cases when a change to a patient’s treatment was needed, GPs would refer patients back to specialist care	Barriers faced by GPs caring for patients with osteoporosis: 1. Competing priorities. Osteoporosis as a comorbidity was often de-prioritised during GP consultations 2. Time and resource limitations; lack of systems and structures to facilitate follow-up and monitoring 3. Poor patient engagement and support for medications due to burdensome regimen and benefits not understood or evident Facilitators to follow-up care included: 1. Personal interest in osteoporosis; one GP with an interest in osteoporosis had established an audited system to routinely follow up patients following their initial prescription. Other GPs did not have such systems, 2. Patient feedback provided by bone health blood marker monitoring	Monitoring blood markers for bone health at commencement and at six months after treatment initiation to provide a structure for follow up and support
Bliuc D, Eisman J, Center JR (2006) [32]		In multivariate analysis, independent predictors of primary care follow-up were age > 50 years (*p* = 0.0003) and patient perception that their fracture was osteoporotic (*p* = 0.03). Among those with a fragility fracture who had a BMD measurement, lower bone density (osteopenia or osteoporosis) was a predictor for GP follow-up (*p* = 0.025)	
Blonk M, Erdtsieck RJ, Wernekinck MGA et al. (2007) [33]		Barriers to GP attendance: 1. Already receiving anti-osteoporotic medication prior to fracture, 2. Refused to go to their GP, 3. Did not understand they were supposed to go to their GP for the prescription and further support	
Casado E, Blanch J, Carbonell C et al. (2021) [42]		Experts identified a lack of effective communication systems between hospital and primary care as a barrier to patient follow-up. Strategies (telephone/email, case manager, shared electronic medical record, standardisation of clinical reports) were suggested to facilitate improved communication but none were considered feasible	
Cranney A, Lam M, Ruhland L et al. (2008) [45]		82% of women reported they had followed up with their primary care physician within 6 months of their fracture Predictors of starting osteoporosis therapy^1^: the intervention (p = 0.002), having a female physician (*p* < 0.005), and baseline osteoporosis knowledge (*p* = 0.015). Predictors of BMD testing: the intervention^1^ (*p* < 0.001) and having a female physician (*p* = 0.032)	Intervention significantly increased: 1. The proportion of women started on prescribed osteoporosis medications (28% (*n* = 35) intervention group versus 10% (*n* = 15) controls, cluster adjusted OR 3.45, 95% CI 1.58–7.56, *p* = 0.002) 2. The proportion of women who had a BMD (53.5% intervention group versus 25.5% controls, cluster adjusted OR 3.38, 95% CI 1.83–6.26, *p* < 0.0001) 3. The proportion of women who received osteoporosis counselling from their PCP (71% vs. 43%, *p* < 0.001)
Drew S, Judge A, Cooper C et al. (2016) [43]	Secondary care clinicians perceived treatment monitoring to be suboptimal in primary care, due to GP knowledge gaps Clinicians did not have confidence that GPs would continue treatments once patients were discharged from the FLS FLS clinicians experienced poor patient care coordination between primary and secondary post-fracture care	Barriers to coordination of osteoporosis care between secondary and primary care: 1. Lack of communication and cooperation between primary and secondary care 2. Insufficient financial incentives to primary care to delivery effective osteoporosis services 3. Variable levels of interest in osteoporosis amongst GPs 4. GP workload pressures	
Inderjeeth CA, Glennon DA, Poland KE et al. (2010) [48]	Most GPs indicated that they reviewed their patients after fracture presentation to the ED, informed them about their risk of osteoporosis, and considered it their responsibility to investigate and manage this risk with the patient. Baseline patient survey data contradicted this, revealing a low level of patient awareness, and a low rate of investigation and treatment uptake	Most GPs indicated they would use simple guidelines, if available	
Jaglal SB, Cameron C, Hawker GA et al. (2006) [49]		Barriers to integrated post-fracture care: 1. Lack of continuity of care 2. Absence of a link between fracture and osteoporosis by patients and HCPs 3. Lack of understanding of GP role in osteoporosis management 4. Patients provided with inconsistent advice regarding follow-up for osteoporosis 5. Poor communication between hospital and primacy care Facilitators to integrated post-fracture care: 1. PCPs wanted to receive reminders/written information about osteoporosis treatment and follow-up care 2. Orthopaedic surgeons discussing osteoporosis with their patients	
Laslett LL, Whitham JM, Gibb C et al. (2007) [31]		Patient barrier to seeing GP following fracture: 1. Being discharged to a rehabilitation centre 2. Residing in a nursing home 3. Belief that they did not need to visit a GP because the fracture had been “fixed”	No between-group significant differences in relation to recommendations for or initiation of osteoporosis investigations or medications
Luc M, Corriveau H, Boire G et al. (2018) [50]		Barriers to adherence to FLS recommendations: 1. Patients receiving inconsistent information (or misinformation) regarding their fracture from HCPs 2. Lack of trust between patient and GP Facilitators to adherence to FLS recommendations: 1. Patients having a clear understanding of their underlying bone disease 2. Telephone follow-up by FLS coordinator was perceived by patients to improve access to healthcare through improved system navigation	
Sale JEM, Bogoch E, Hawker G et al. (2014) [44]		Provider-level barriers to post-fracture care experienced by patients before fracture risk assessment: 1. Deeming the fracture to be either an isolated incident or the result of more severe trauma 2. Assuming the patient had normal bone density based on his or her appearance or demographic characteristic 3. Advising patients that BMD testing was not appropriate in their circumstances 4. Using x-rays to make judgements about bone density Provider-level barriers to post-fracture care experienced by patients after fracture risk assessment: 1. Lack of communication by GP about the results of fracture risk assessment 2. Delivery of incorrect information about bone health treatment by GP	
Vaculik J, Stepan JJ, Dungl P et al. (2017) [47]			Compared with general recommendations, providing patients and their GPs with detailed individualised recommendations for osteoporosis investigation and management following fracture did not lead to an increase in use of BMD testing, x-ray imaging, specialist care, or prescription for anti-osteoporosis medication
Zinger G, Sylvetsky N, Levy Y et al. (2021) [46]			Significantly more patients in group 1 (type B FLS with monthly telephone coaching) were receiving recommended treatment^1^ at 4 months, compared with those in group 2 (type C FLS) (77.1% vs 6.2%, *p* < 0.0001)

*HCP* healthcare practitioner; *PCP* primary care physician, synonymous with general practitioner (GP); *GP* general practitioner; *25(OH)D* 25-hydroxyvitamin D

1. Starting osteoporosis therapy or performing bone health investigations (having BMD or 25(OH)D measured) were considered indicators of primary care follow-up in studies where these activities were the responsibility of the GP

### Results of synthesis

Supplement 5 provides a summary of the thematic analysis described in detail below.

1. Research question 1: experiences of the FLS-to-primary care transition

Among the four studies reporting findings relevant to the experience of the FLS-to-primary care transition, five key themes emerged: (1) time and workload pressures, (2) limited confidence in primary care follow-up, (3) GP knowledge gaps, (4) siloed or disconnected care, and (5) communication issues. Overall, analysis was limited by the small number of studies and the small sample size of each study.

Theme one was the most consistent theme and was supported by three studies [40, 41, 43], all of which were qualitative studies of moderate-to-high methodological quality with low risk of bias. While the FLS-to-primary care transition was the principal focus of one of these studies [40], the remaining studies focused on specific components of the FLS model (Bishop et al. explored prescribing experience, and Drew et al. explored components of post-hip fracture care), which provided insights into the perceptions of the healthcare transition from the perspectives of healthcare practitioners. Time and workload pressures were perceived to contribute to suboptimal post-fracture care; however, this phenomenon was only reported with reference to primary care and no data alluded to similar pressures in the FLS setting.

Two studies [40, 41] reported data from FLS clinicians highlighting their limited confidence in primary care to deliver optimal post-fracture care (theme 2). These concerns appeared to stem from an awareness of: GP time and workload pressures (theme 1), GP knowledge gaps (theme 2), and communication barriers (theme 5) arising from a disconnection between the FLS and primary care (theme 4). Bishop et al. found that several GPs experienced uncertainty with regard to osteoporosis management arising from gaps in knowledge, in particular determining the optimal duration of bisphosphonate therapy and interpreting results (e.g. bone mineral density reports and fracture risk assessment scores). Advice from a secondary care specialist (e.g. FLS clinician) was perceived as helpful in addressing the learning needs of some GPs; however, this was not universally accessible [41]. Two studies [40, 43] detailed clinician experience of acute/FLS care as siloed or disconnected from primary care, leading to poor communication and discontinuity of patient care. One study [48] of low quality and high risk of bias, identified discordance between GPs’ and patients’ experiences of GP-led post-fracture follow-up. In particular, most GPs reported that they reviewed their patients following an hospital presentation with fracture and informed them of their fracture risk; however, patient survey data indicated a low level of patient awareness and uptake of bone health investigations and treatment.

2. Research question 2: barriers and facilitators to PC follow-up after FLS

Barriers and facilitators to primary care follow-up after FLS fell into five themes: (1) patient knowledge and understanding, (2) miscommunication and misinformation, (3) understanding roles and responsibilities, (4) GP engagement, and (5) GP-patient relationship. Single studies suggested healthcare policies and funding, accessing primary care from residential facilities, and GP gender were additional influencing factors; however, these were not considered themes due to insufficient data.

High-quality [32, 41, 45] and moderate-quality [33, 40, 49, 50] evidence supported patient knowledge and understanding (theme 1) as a leading factor affecting primary care follow-up after FLS. In particular, patients needed to appreciate the link between their fracture and osteoporosis (subtheme 1), appreciate the seriousness of the diagnosis (subtheme 2), and understand follow-up recommendations (subtheme 3). Subtheme 1 was supported by data from two randomised controlled trials [32, 45] and one mixed-methods study [50]; Bliuc et al. reported patients’ perception of their fracture as osteoporotic as an independent predictor of GP follow-up [32], Cranney et al. found baseline osteoporosis knowledge significantly predicted starting osteoporosis therapy [45], and Luc et al. identified patient recognition of fragility fracture as a sign of underlying bone disease as one of three main themes influencing adherence to FLS recommendations [50]. Subtheme 2 was supported by triangulation of data from multiple qualitative studies [40, 41, 49], whereas subtheme 3 was supported by triangulation of data from quantitative [31, 33], mixed-methods [49], and qualitative studies [40].

Miscommunication and misinformation by healthcare providers (theme 2) emerged as a threat to patient acquisition of knowledge and understanding of their condition. In their analysis of provider-level barriers to post-fracture secondary prevention (high quality, low risk of bias), Sale et al. identified two key themes following fracture risk assessment: a lack of communication about fracture risk and misinformation about treatment [44]. GPs’ misconceptions about fracture risk led to patients being misinformed about their fracture risk [44]. This prevented patients from appreciating the link between fracture and osteoporosis, appreciating the seriousness of their condition, being offered treatment, and responding with appropriate health behaviours. Another study (moderate quality, moderate risk of bias) reported inconsistent information about fracture risk by various healthcare providers as a leading barrier to patient adherence to FLS recommendations [50].

Poor understanding of the roles and responsibilities of FLS clinicians and GPs (theme 3) was reported by three studies [40, 41, 48]. Bennett et al. found that GPs identified “understanding roles and responsibilities” as a central theme affecting post-FLS care. While GPs in several studies self-identified themselves as chiefly responsible for osteoporosis care [40, 48, 49], their awareness of the role of the FLS was deemed as low in one qualitative study [40]. This contrasted the view of patients, who perceived FLS clinicians (as opposed to GPs) to be responsible for their long-term osteoporosis care [40].

Moderate- and high-quality evidence suggested that GP engagement is required for FLS treatment recommendations to be continued in primary care. Sale et al. found that only 2 of 18 patients attending their GP following a bone mineral density test discussed fracture risk [44]. Three subthemes emerged influencing GP engagement: (1) willingness to manage osteoporosis, (2) prioritising osteoporosis management, and (3) available resources. Despite the aforementioned concerns about GP knowledge gaps [41] and misconceptions about fracture risk [44], Bennett et al. reported GPs to be confident and willing to lead patients’ long-term post-fracture care [40] and Inderjeeth et al. found that most GPs felt they would use osteoporosis guidelines to support management, if available [48]. Two moderate-quality studies [40, 49] suggested that low levels of GP awareness of osteoporosis and competing medical priorities were barriers to prioritising osteoporosis management; however, these studies were small (collectively included only 37 GPs), potentially limiting the transferability of findings. Resource limitation (in particular, time and workload pressures—see also RQ1, above) was identified as a barrier to GP engagement in follow-up activities in two small qualitative studies [41, 43].

Qualities of the GP-patient relationship (theme 5) appeared to influence patient adherence to recommendations made by the FLS. Qualitative patient data from two moderate-quality studies indicated patient trust and confidence in their GP supported medication adherence [50] and GP attendance [40]. Conversely, a lack of trust adversely affected patient engagement.

Single studies indicated that female GP gender may predict GP follow-up for FLS patients [45], whereas a lack of financial incentives for delivering osteoporosis care in primary care [41], and difficulties accessing GPs from residential care facilities [31] were barriers to care. Further analysis of these factors was not possible due to limited data.

3. Research question 3: interventions to enhance integration

Five studies included interventions applied with the aim of enhancing integration of hospital-based secondary fracture prevention services with primary care [31, 41, 45–47]. Interventions included blood marker monitoring [41], a clinical protocol for low-trauma fractures [31], detailed individualised (vs general) management recommendations for GPs [47], telephone coaching [46], and a multifaceted intervention of patient and clinician reminders and education [45]. Studies exploring individual vs. general treatment recommendations [47] or a clinical protocol for low trauma fractures [31] did not demonstrate a significant intervention effect. Both studies were of low methodological quality and had a high risk of bias.

Cranney et al. detailed a multifaceted intervention comprised of patient and provider reminders and educational material [45]. Postmenopausal women (*n* = 270) with fragility wrist fracture were recruited from hospital emergency departments and randomised to intervention (*n* = 125) or usual care (*n* = 145) groups. Intervention participants had a personalised letter sent to their GP detailing their recent fracture, a recommendation to investigate for osteoporosis and supporting educational material (a summary of recommended therapies and a treatment algorithm). Similarly, patients were sent a letter at two weeks and reminder at two months post-fracture. The letter contained educational material (fracture risk checklist, educational booklet, and summary of treatment options) and a recommendation to discuss osteoporosis with their GP. Compared with usual care, significantly more women in the intervention group reported starting osteoporosis medication (28% vs. 10%, *p* = 0.002) and receiving osteoporosis counselling from their GP (71% vs. 43%, *P* < 0.001); however, there was no significant difference in the number of women who reported being told their fracture was due to osteoporosis and there was no significant improvement in osteoporosis knowledge score (OPQ) from baseline to 6-month follow-up. In many respects, the study interventions could be considered standard care under a type C FLS model (identify patients at risk of osteoporosis and inform them and their GP) [16]; however, the content of GP education and use of patient reminder was considered novel. While their multifaceted intervention improved indicators of care coordination (medication initiation and GP discussion), it remains unclear how much of this effect is attributable to the novel components. The lack of significant change in OPQ scores suggests the educational components may not have been effective.

In their qualitative study of 23 clinicians’ views of prescribing bisphosphonates for osteoporosis, Bishop et al. included data from one clinician who represented a blood marker monitoring service (presumably measuring markers of bone turnover), which is marketed to patients as an alternative to bone mineral density testing [41]. The service was perceived to offer patients feedback on the effect of their medication, providing structure and support to follow-up. Limitations of this study include small sample size (only one participant from the bone marker monitoring service was interviewed) and potential bias (the participant is described as “representing” the bone marker monitoring service and financial conflicts are not listed).

One study (high quality, moderate risk of bias) compared patient-reported treatment initiation rates 4-months post-fracture with two interventions [46]. The first intervention involved a patient letter, addressed to their GP recommending their GP start medication for osteoporosis (letter group). The second intervention involved four components: printed patient information, a bone mineral density scan with a report addressed to the GP, a specific treatment recommendation, and monthly phone calls encouraging patients to commence treatment. The letter intervention can be considered a type C FLS, whereas the intervention group can be considered a type B FLS with the addition of telephone coaching. The authors reported significantly more patients in the intervention group received recommended treatment at 4 months, compared with the letter group (77.1% vs. 6.2%, *P* < 0.0001). As the two study interventions differed in multiple ways, reported effects cannot be solely attributed to the telephone coaching component of the intervention. Moreover, patient-reported outcomes are a potential source of bias and the authors do not report the proportion of patients in each group who discussed recommendations with their GP, only those who were taking treatment 4 months post-fracture.

### Certainty of evidence

While key themes were extracted from available studies, it was not possible to draw firm conclusions or quantify outcomes relevant to these research questions due to the paucity of studies, many of which did not specifically address these research questions and contained methodological limitations affecting confidence in their findings. The results of data synthesis largely reflect the outcomes of small qualitative and mixed-methods studies that contain limited details regarding participant selection and context. The outcomes of qualitative studies reflect the experiences of individual participants, which were influenced by their interactions with their environments (including researchers), as well as their attitudes, beliefs, and opinions. Included studies were performed in a range of healthcare settings across various countries and experiences are likely to differ between settings, all of which limited conclusions.

## Discussion

### General interpretation of results

Thematic analysis depicted the experience of the FLS-to-primary care transition for patients and HCPs as challenging and disjointed, affected by (1) GP time and workload pressures, (2) limited FLS clinician confidence in primary care follow-up, (3) GP knowledge gaps, (4) siloed or disconnected care, and (5) communication issues. Data from multiple studies with principally qualitative methodologies suggested the following factors affect primary care follow-up after FLS: (1) patient knowledge and understanding, (2) miscommunication and misinformation, (3) understanding of roles and responsibilities, (4) GP engagement, and (5) quality of the GP-patient relationship. A single study suggested that healthcare policies and funding may be additional influencing factors. GP education and patient reminders, delivered together as part of a multifaceted intervention in the setting of a type C FLS appeared to improve the integration of acute and primary care post-fracture care. While telephone coaching and bone marker monitoring were identified as potential interventions to enhance FLS integration with primary care, there was insufficient evidence to conclude they were effective.

Overall, the FLS to primary care transition appears to be a challenging period for patients, GPs, and FLS clinicians who experience multiple barriers to care continuity at the patient, provider, and system level. Many of the themes reported may not be uniquely attributable to the FLS-to-primary care transition but reflect poor *healthcare integration*, a term used to describe the management and delivery of different health services across different levels within the healthcare system. Commonly reported barriers to healthcare integration, with links to this analysis include time pressures and staffing levels, professional engagement, insufficient communication, and role confusion [51]. Conversely, several factors that may affect healthcare integration have not yet been reported in the FLS-to-primary care setting suggesting research on this topic is incomplete: patient/caregiver engagement, preparation for healthcare transition, HCP collaboration, treatment complexity, and social challenges [52, 53].

In the four studies exploring stakeholders’ experience of the FLS to primary care transition, emergent themes reflected the many difficulties patients and clinicians face moving and working across disparate services. To understand what factors might facilitate a more seamless healthcare transition, Baxter et al. conducted a novel study exploring the experiences of staff working within general practices, hospital teams (aged care, cardiology, respiratory) and affiliated community care teams, all characterised by very low hospital readmission rates, a widely accepted indicator of overall quality care [54]. Analysis of the attributes of these high-performing teams identified three key themes: knowing the patient, knowing each other, and bridging gaps. Staff built trust and rapport with patients and gathered a holistic and shared understanding of patients’ needs. They developed relationships within and across teams and bridged care gaps by enhancing communication, adjusting patient expectations, and adapting to changing services or priorities. The paper by Baxter et al. offers a case study for well-integrated care and by focussing on facilitators to care coordination they provide a counter perspective to the barriers-focussed data reviewed here.

There was insufficient evidence to support any single intervention (beyond standard FLS interventions) for enhancing care coordination between FLS and primary care; however, GP education and patient reminders improved markers of care coordination when delivered together by a service analogous to a type C FLS. Examples of other successful interventions can be found in the broader literature, which may have potential application to the FLS-primary care setting. The World Health Organization (WHO) identifies a range of interventions to support various forms of care continuity and coordination in their Framework on Integrated People-Centred Health Services (IPCHS) [55]; shared electronic medical records and technology-enabled care support *information continuity*; interdisciplinary team-based practices, colocation of services, clinical networks, and care pathways/guidelines support *team continuity*; health promotion, collaborative care planning, tailored health literacy, and self-management coaching support *flexible continuity*; patient-centred medical homes, health navigators, and case managers support *longitudinal continuity*; and peer support, social networks, community health agents, and workforce education support *interpersonal continuity*. Current best practice models of FLS include some interventions to support continuity, including patient education, provision of a long-term management plan to GPs, and short-term (often telephone based) follow-up to assess adherence. However, such interventions have not been universally adopted and there is evidence of unwarranted variation in service delivery between FLS [40, 56]. Given the heterogeneity of services and evidence of multiple residual barriers to primary-care follow-up, a systematic approach is needed to address barriers and enhance facilitators to care continuity and coordination.

A recent review of osteoporosis integrated care initiatives over the past decade found 69 unique initiatives had been studied with the aim of improving collaboration between HCPs, departments, and health sectors [57]. Initiatives were classified according to the strategies and substrategies of the WHO IPCHS framework [58]. While many studies involved coordinating care for individuals using interventions that are now considered standard elements of the FLS model (such as the use of a care coordinator, team-based care, and standardised assessments), no studies reported interventions for improving coordination between health care providers (IPCHS strategy 4.2: coordinating health programs and providers) or coordinating with primary care upon discharge (IPCHS strategy 3.3: building strong primary care based systems).

Of particular concern to the FLS-primary care transition is ensuring osteoporosis medications are continued so that their anti-fracture benefits are sustained. Long-term persistence with osteoporosis medications after fracture is poor [24] even in the setting of a gold standard FLS [25]. A recent systematic review and meta-analysis by Tomlinson et al. found that among older people discharged from hospital, interventions that successfully support medication continuity include those with components of self-management, telephone follow-up, or medication reconciliation [59]. However, the durability of continuity of care initiatives has been questioned by a 2020 meta-analysis by Facchinetti et al., who found inconclusive evidence for long-term effectiveness (as assessed by 3–6-month re-admission rates) [60]. It is worth noting that none of the studies selected patient populations with osteoporosis. Research exploring transitions of care for osteoporosis patients is scant; however, an ethnographic study by Stolee et al. identified six potential intervention points for improving the healthcare transition of post-acute hip fracture patients: patient involvement and choice, family caregiver roles, strong relationships, coordination of roles, documentation, and information sharing [61]. There is a need for further research to investigate novel interventions targeting these areas across the FLS to primary care transition.

### Limitations

Overall, this analysis was limited by the small number of included studies, most of which did not directly address the specific review questions, and many of which were affected by small sample size, low methodological quality, and moderate-to-high risk of bias. The themes arising from this analysis are predominantly focussed on the micro (providers) or meso (intervention) level and it is possible that unidentified system level or external contextual factors may be present but not captured in available evidence. Moreover, most studies reported challenges or barriers to care, and studies reporting positive stakeholder experience and facilitators to care may have been affected by publication bias. By limiting the search to English-language studies, relevant studies published in other languages may have been missed.

The review search strategy adopted a broad definition of *FLS*, and many studies did not explicitly describe their service or intervention as an FLS; however, studies were included if their intervention or activities functioned as such.

JBI critical appraisal tools were chosen for quality assessment as they offer a range of checklists tailored to diverse study methodologies; however, several studies did not conform to JBI checklist requirements (e.g. quantitative non-randomised studies and mixed-methods studies), and a “best fit” approach was taken to selecting the most appropriate tool for appraisal. In some instances, multiple tools were used, with the final quality score determined through joint reviewer assessment and discussion.

The authors acknowledge the inherently subjective nature of thematic analysis and employed strategies of reflexivity and independent analysis to manage this.

## Conclusions and implications

Our results suggest that barriers to care continuity and coordination exist across the FLS-to-primary care transition and addressing these could be the subject of future research. Knowledge gaps exist regarding the patient experience of the care transition, facilitators to primary care follow-up, and interventions to support FLS integration with primary care. There is a paucity of evidence concerning patient experience of the FLS to PC transition. Ensuring diverse patient/consumer engagement should be a priority for future studies in this area. Overall, current data portray a predominantly negative picture of the FLS to PC transition, characterised by multiple barriers to care continuity. Further research could focus on facilitators to continuity and coordination of care within the post-FLS context. While single studies suggested healthcare polices and funding, GP gender, and accessing primary care from residential facilities may influence GP follow-up after FLS, future research could investigate this further.

High-quality data are needed to evaluate the impact of interventions designed to coordinate care between FLS and primary care. Before this can be achieved, it would help to have one or more agreed measures of successful integration. Within the transitional care literature, hospital readmission (in particular, potentially avoidable readmission within 28 or 30 days of discharge) has served as a gold standard quality metric, but this may not be the most suitable measure of successful care transition in the post-FLS setting. Further, in Australia, a re-fracture due to untreated osteoporosis is not recognised as an avoidable hospital readmission [62]. It may be useful to consider other outcomes, such as medication persistence at defined time points, patient-centred measures such as the care transition measure, or bundled measures. Standardised outcome measures will then allow researchers and policymakers to compare effectiveness of different interventions and benchmark individual services.

## Supplementary Information

Below is the link to the electronic supplementary material.

ESM 1 (DOCX 251 KB)
