# Supplementary material for: Primary care follow-up of patients after attending a fracture liaison service: an integrative review
Source: Arch Osteoporos. 2025 May 13;20(1):65. doi: 10.1007/s11657-025-01521-8 (PMC12075282; doi:10.1007/s11657-025-01521-8)
Supplement: Supplementary file 1 — (DOCX 251 KB) [file 11657_2025_1521_MOESM1_ESM.docx]

**Supplementary information**

**SUPPLEMENT 1**

**Table**: Search strategy and search terms.

| **Database** | **Concept** | **Search terms** | **Results** |
| --- | --- | --- | --- |
| **CINAHL**  (EBSCO*Host*)  (1 January 2003 to 29 December 2023) | #1 | "Fragility fracture*" OR "osteoporosis" OR "Reduced bone density" OR "low bone density" OR "hip fracture*" OR "spine fracture*" OR "vertebral fracture*" OR "minimal trauma fracture*" OR "minimal-trauma fracture*" OR “fracture” | 6,509 |
|  | #2 | "Primary care*" OR "primary health care*" OR "primary healthcare*" OR "general practice*" OR "general practitioner*" OR "primary health care* provider*" OR "Primary healthcare provider*" OR "primary health-care provider" OR "community care" OR "community service*" OR "family physician*" OR "family practitioner*" OR "family medicine" | 19,040 |
|  | #3 | "refracture* prevention" OR "re-fracture* prevention" OR "fracture* liaison*" OR "secondary fracture* prevention" OR "secondary fracture*" OR "secondary care*" OR "osteoporosis refracture prevention" OR "osteoporosis clinic*" OR "fracture* clinic*" OR “metabolic bone* clinic*" OR "bone* clinic*" | 516 |
|  | #4 | #1 AND #2 AND #3 | **326** |
|  | Filters | Human participants  Research article  Adults aged 45-64 years  Adults aged 65+ years  English language  Search all text fields  Exclude MEDLINE records |  |
| **EMBASE** (Ovid)  (1 January 2003 to 29 December 2023) | #1 | osteoporosis/ or corticosteroid induced osteoporosis/ or idiopathic osteoporosis/ or postmenopause osteoporosis/ or primary osteoporosis/ or secondary osteoporosis/ or senile osteoporosis/ or fracture/ or fragility fracture/ or limb fracture/ or multiple fracture/ or pelvis fracture/ or rib fracture/ or spine fracture/ or hip fracture/ or "reduced bone densit*".mp. or "low bone densit*".mp. or "minimal trauma fracture*".mp. or "minimal-trauma fracture*".mp | 87,948 |
|  | #2 | primary medical care/ or primary health care/ or general practitioner/ or family medicine/ or general practice/ or "primary care*".mp. or "primary healthcare*".mp. or "community care".mp. or "community service*".mp. or "family physician*".mp. or "family practitioner*".mp | 121,716 |
|  | #3 | "refracture prevention" or "re-fracture prevention" or "fracture liaison" or "secondary fracture prevention" or "secondary fracture*" or "secondary care*" or "osteoporosis refracture prevention" or "osteoporosis re-fracture prevention" or "osteoporosis clinic*" or "fracture clinic*" or "metabolic bone clinic*" or "bone clinic*".mp | 10,232 |
|  | #4 | #1 AND #2 AND #3 | **322** |
|  | Filters | Keyword search  Map term to subject heading  English language  Human participants  Remove MEDLINE records |  |
| **PubMed**  (1 January 2003 to 29 December 2023) | #1 | “Osteoporosis, Postmenopausal"[Mesh] OR "Osteoporosis"[Mesh] OR "Osteoporotic Fractures"[Mesh] OR Osteoporosis[tw] OR “fragility fracture*”[tw] OR “reduced bone density”[tw] OR “low bone density”[tw] OR “hip fracture*”[tw] OR “spine fracture*”[tw] OR “spinal fracture*”[tw] OR “vertebral fracture*”[tw] OR “minimal trauma fracture*”[tw] OR “minimal-trauma fracture*”[tw] | 74,812 |
|  | #2 | "Primary Health Care"[Mesh] OR “primary care”[tw] OR “primary health care”[tw] OR “primary healthcare”[tw] OR “general practice*”[tw] OR “general practitioner*”[tw] OR “primary health care provider*”[tw] OR “primary healthcare provider*”[tw] OR “primary health-care provider*”[tw] OR “primary health provider*”[tw] OR “primary health service*”[tw] OR “community care*”[tw] OR “community service*”[tw] OR “family physician*”[tw] OR “family practitioner*”[tw] OR “family medicine”[tw] | 239,797 |
|  | #3 | "Secondary Prevention"[Mesh] OR “refracture prevention*”[tw] OR “re-fracture prevention*”[tw] OR “fracture liaison*”[tw] OR “secondary fracture prevention*”[tw] OR “secondary fracture*”[tw] OR “secondary care*”[tw] | 25,293 |
|  | #4 | #1 AND #2 AND #3 | **128** |
|  | Filters | Text words [TW] search  Human participants  Adults aged 45+ years  English language  Search all fields |  |
| **Social Sciences Citation Index** (Web of Science)  (1 January 2003 to 29 December 2023) | #1 | "Fragility fracture*" OR "osteoporosis" OR "Reduced bone density" OR "low bone density" OR "hip fracture*" OR "spine fracture*" OR "vertebral fracture*" OR "minimal trauma fracture*" OR "minimal-trauma fracture*" OR “fracture” | 382,865 |
|  | #2 | "Primary care*" OR "primary health care*" OR "primary healthcare*" OR "general practice*" OR "general practitioner*" OR "primary health care* provider*" OR "Primary healthcare provider*" OR "primary health-care provider" OR "community care" OR "community service*" OR "family physician*" OR "family practitioner*" OR "family medicine" | 258,262 |
|  | #3 | "refracture* prevention" OR "re-fracture* prevention" OR "fracture* liaison*" OR "secondary fracture* prevention" OR "secondary fracture*" OR "secondary care*" OR "osteoporosis refracture prevention" OR "osteoporosis clinic*" OR "fracture* clinic*" OR “metabolic bone* clinic*" OR "bone* clinic*" | 8,911 |
|  | #4 | #1 AND #2 AND #3 | **184** |
|  | Filters | English language  Document type: Article  Search all fields |  |
| **Applied Social Sciences Index and Abstracts**  (ProQuest)  (1 January 2003 to 29 December 2023) | #1 | noft("fragility fracture*") OR noft("osteoporosis") OR noft("reduced bone density") OR noft("low bone density") OR noft("hip fracture*") OR noft("spine fracture*") OR noft("vertebral fracture*") OR noft("minimal trauma fracture*") OR noft("minimal-trauma fracture*") OR noft(“fracture”) | 290,195 |
|  | #2 | noft("Primary care*") OR noft("primary health care*") OR noft("primary healthcare*") OR noft("general practice*") OR noft("general practitioner*") OR noft("primary health care* provider*") OR noft("Primary healthcare provider*") OR noft("primary health-care provider") OR noft("community care") OR noft("community service*") OR noft("family physician*") OR noft("family practitioner*") OR noft("family medicine") | 208,240 |
|  | #3 | “refracture* prevention” OR “re-fracture* prevention” OR “fracture* liaison*” OR “secondary fracture* prevention” OR “secondary fracture*” OR “secondary care*” OR “osteoporosis refracture prevention” OR “osteoporosis clinic*” OR “fracture* clinic*” OR “metabolic bone* clinic*” OR “bone* clinic*” | 7,971 |
|  | #4 | #1 AND #2 AND #3 | **174** |
|  | Filters | Search anywhere  English language  Document type: Article  Peer reviewed  Source type: scholarly journals  Exclude Medline |  |
| **Combined** |  | Total | **1,134** |

**SUPPLEMENT 2**

**Table**: Results of risk of bias assessment using the Mixed Methods Appraisal Tool (MMAT).

| **Manuscript** | **Overall score** | **MMAT Criteria** | **Yes** | **No** | **Can’t tell** |
| --- | --- | --- | --- | --- | --- |
| Bennett MJ, Center JR, Perry L (2023) [40] | 100 | Is the qualitative approach appropriate to answer the research question? | X |  |  |
|  |  | Are the qualitative data collection methods adequate to address the research question? | X |  |  |
|  |  | Are the findings adequately derived from the data? | X |  |  |
|  |  | Is the interpretation of results sufficiently substantiated by data? | X |  |  |
|  |  | Is there coherence between qualitative data sources, collection, analysis and interpretation? | X |  |  |
| Bishop S, Narayanasamy MJ, Paskins Z et al (2023) [41] | 100 | Is the qualitative approach appropriate to answer the research question? | X |  |  |
|  |  | Are the qualitative data collection methods adequate to address the research question? | X |  |  |
|  |  | Are the findings adequately derived from the data? | X |  |  |
|  |  | Is the interpretation of results sufficiently substantiated by data? | X |  |  |
|  |  | Is there coherence between qualitative data sources, collection, analysis and interpretation? | X |  |  |
| Bliuc D, Eisman J, Center JR (2006) [32] | 50 | Is randomization appropriately performed? | X |  |  |
|  |  | Are the groups comparable at baseline? | X |  |  |
|  |  | Are there complete outcome data? |  | X |  |
|  |  | Are outcome assessors blinded to the intervention provided? |  |  | X |
|  |  | Did the participants adhere to the assigned intervention? | X |  |  |
| Blonk M, Erdtsieck RJ, Wernekinck MGA et al (2007) [33] | 50 | Is the sampling strategy relevant to address the research question? | X |  |  |
|  |  | Is the sample representative of the target population? | X |  |  |
|  |  | Are the measurements appropriate? |  |  | X |
|  |  | Is the risk of nonresponse bias low? | X |  |  |
|  |  | Is the statistical analysis appropriate to answer the research question? |  |  | X |
| Casado E, Blanch J, Carbonell C et al (2021) [42] | 100 | Is the qualitative approach appropriate to answer the research question? | X |  |  |
|  |  | Are the qualitative data collection methods adequate to address the research question? | X |  |  |
|  |  | Are the findings adequately derived from the data? | X |  |  |
|  |  | Is the interpretation of results sufficiently substantiated by data? | X |  |  |
|  |  | Is there coherence between qualitative data sources, collection, analysis and interpretation? | X |  |  |
| Cranney A, Lam M, Ruhland L et al (2008) [45] | 75 | Is randomization appropriately performed? | X |  |  |
|  |  | Are the groups comparable at baseline? | X |  |  |
|  |  | Are there complete outcome data? | X |  |  |
|  |  | Are outcome assessors blinded to the intervention provided? |  |  | X |
|  |  | Did the participants adhere to the assigned intervention? | X |  |  |
| Drew S, Judge A, Cooper C et al (2016) [43] | 100 | Is the qualitative approach appropriate to answer the research question? | X |  |  |
|  |  | Are the qualitative data collection methods adequate to address the research question? | X |  |  |
|  |  | Are the findings adequately derived from the data? | X |  |  |
|  |  | Is the interpretation of results sufficiently substantiated by data? | X |  |  |
|  |  | Is there coherence between qualitative data sources, collection, analysis and interpretation? | X |  |  |
| Inderjeeth CA, Glennon DA, Poland KE et al (2010) [48] | 25 | Is the sampling strategy relevant to address the research question? | X |  |  |
|  |  | Is the sample representative of the target population? |  |  | X |
|  |  | Are the measurements appropriate? |  |  | X |
|  |  | Is the risk of nonresponse bias low? |  | X |  |
|  |  | Is the statistical analysis appropriate to answer the research question? |  | X |  |
| Jaglal SB, Cameron C, Hawker GA et al (2006) [49] | 100 | Is there an adequate rationale for using a mixed methods design to address the research question? | X |  |  |
|  |  | Are the different components of the study effectively integrated to answer the research question? | X |  |  |
|  |  | Are the outputs of the integration of qualitative and quantitative components adequately interpreted? | X |  |  |
|  |  | Are divergences and inconsistencies between quantitative and qualitative results adequately addressed? | X |  |  |
|  |  | Do the different components of the study adhere to the quality criteria of each tradition of the methods involved? | X |  |  |
| Laslett LL, Whitham JM, Gibb C et al (2007) [31] | 50 | Are the participants representative of the target population? | X |  |  |
|  |  | Are measurements appropriate regarding both the outcome and intervention (or exposure)? |  |  | X |
|  |  | Are there complete outcome data? |  | X |  |
|  |  | Are the confounders accounted for in the design and analysis? |  |  | X |
|  |  | During the study period, is the intervention administered (or exposure occurred) as intended? | X |  |  |
| Luc M, Corriveau H, Boire G et al (2018) [50] | 75 | Is there an adequate rationale for using a mixed methods design to address the research question? | X |  |  |
|  |  | Are the different components of the study effectively integrated to answer the research question? | X |  |  |
|  |  | Are the outputs of the integration of qualitative and quantitative components adequately interpreted? | X |  |  |
|  |  | Are divergences and inconsistencies between quantitative and qualitative results adequately addressed? |  |  | X |
|  |  | Do the different components of the study adhere to the quality criteria of each tradition of the methods involved? | X |  |  |
| Sale JEM, Bogoch E, Hawker G et al (2014) [44] | 100 | Is the qualitative approach appropriate to answer the research question? | X |  |  |
|  |  | Are the qualitative data collection methods adequate to address the research question? | X |  |  |
|  |  | Are the findings adequately derived from the data? | X |  |  |
|  |  | Is the interpretation of results sufficiently substantiated by data? | X |  |  |
|  |  | Is there coherence between qualitative data sources, collection, analysis and interpretation? | X |  |  |
| Vaculik J, Stepan JJ, Dungl P et al (2017) [47] | 25 | Are the participants representative of the target population? | X |  |  |
|  |  | Are measurements appropriate regarding both the outcome and intervention (or exposure)? |  | X |  |
|  |  | Are there complete outcome data? |  | X |  |
|  |  | Are the confounders accounted for in the design and analysis? |  |  | X |
|  |  | During the study period, is the intervention administered (or exposure occurred) as intended? |  | X |  |
| Zinger G, Sylvetsky N, Levy Y et al (2021) [46] | 50 | Is randomization appropriately performed? | X |  |  |
|  |  | Are the groups comparable at baseline? |  |  | X |
|  |  | Are there complete outcome data? | X |  |  |
|  |  | Are outcome assessors blinded to the intervention provided? |  | X |  |
|  |  | Did the participants adhere to the assigned intervention? |  |  | X |

FLS; Fracture liaison service

BMD; Bone mineral density

GP; General practitioner

**SUPPLEMENT 3**

**Table**: Results of quality assessment using the Joanna Briggs Institute (JBI) Critical Appraisal Tools.

| **Author (year)** | **Design** | **JBI Checklist Question** | | | | | | | | | | | | | **Overall quality score^1^** | **Notes** |
| --- | --- | --- | --- | --- | --- | --- | --- | --- | --- | --- | --- | --- | --- | --- | --- | --- |
|  |  | **1** | **2** | **3** | **4** | **5** | **6** | **7** | **8** | **9** | **10** | **11** | **12** | **13** |  |  |
| Bennett MJ, Center JR, Perry L (2023) [40] | Qualitative study | U | Y | Y | Y | Y | N | N | Y | Y | Y | N/A | N/A | N/A | Moderate | Philosophical perspective used by the authors is not stated. |
| Bishop S, Narayanasamy MJ, Paskins Z et al (2023) [41] | Qualitative study | Y | Y | Y | Y | Y | N | N | Y | Y | Y | N/A | N/A | N/A | High | Only one clinician from the bone marker monitoring service was included. They are described as “representing” the service and their financial arrangements with the service are not stated, raising the possibility of bias. |
| Bliuc D, Eisman J, Center JR (2006) [32] | Quantitative randomised trial | Y | Y | Y | N | N | Y | N | Y | Y | Y | Y | Y | Y | High | JBI tool for RCT used as there is no JBI tool for quantitative randomised trials. Outcomes of this study were patient-reported via telephone interview. For the purpose of quality assessment this was considered a “reliable” way of measuring the outcomes. |
| Blonk M, Erdtsieck RJ, Wernekinck MGA et al (2007) [33] | Quantitative descriptive study | Y | Y | Y | U | N | Y | Y | Y | Y | Y | N/A | N/A | N/A | Moderate | JBI tool for case series studies used as there is no JBI tool for quantitative descriptive studies. While the JBI score suggests it is a high-quality study, we have indicated it to be moderate quality due to a number of issues: the contents of the 3-month compliance questionnaire is not listed, there is a moderate non-response rate, and the study relies solely on patient self-reported outcome measures. |
| Casado E, Blanch J, Carbonell C et al (2021) [42] | Qualitative (Delphi) study | N/A | Y | Y | Y | Y | N/A | N/A | Y | N | Y | N/A | N/A | N/A | Moderate | While the total score (85%) indicates high quality, there is no JBI tool for Delphi Studies and the “Qualitative Study” tool includes several criteria that are not applicable. Moreover, no patient/consumer representative was included in the expert panel and it is unclear whether ethical approval was sought for this study. I have therefore graded the quality as “moderate”. |
| Cranney A, Lam M, Ruhland L et al (2008) [45] | Quantitative randomised trial | Y | Y | Y | N | N | Y | Y | Y | Y | Y | Y | Y | Y | High | JBI tool for RCT used. For the purpose of quality assessment this was considered a reliable way of measuring the outcomes. |
| Drew S, Judge A, Cooper C et al (2016) [43] | Qualitative study | U | Y | Y | Y | Y | N | N | Y | Y | Y | N/A | N/A | N/A | Moderate | Philosophical perspective used by the authors is not stated. |
| Inderjeeth CA, Glennon DA, Poland KE et al (2010) [48] | Quantitative descriptive study | Y | Y | U | U | Y | U | Y | N | U | N/A | N/A | N/A | N/A | Low | JBI tool for quasi-experimental studies used. Baseline characteristics of patients not included. Limited details provided on the historical comparison group. Statistical tests used for comparisons are not detailed. Participant survey questions not detailed. Low patient response rate may have biased outcome. |
| Jaglal SB, Cameron C, Hawker GA et al (2006) [49] | Mixed methods study | U | Y | Y | Y | Y | N | N | N | Y | Y | N/A | N/A | N/A | Moderate | As this is principally a qualitative study, the JBI tool for qualitative studies used for quality assessment. Lack of quotes to support themes arising from focus groups. |
| Laslett LL, Whitham JM, Gibb C et al (2007) [31] | Quantitative non-randomised study | Y | Y | N | Y | Y | Y | N | N | Y | N/A | N/A | N/A | N/A | Low | JBI tool for quasi-experimental studies used. Significant differences between groups noted. No mention of response rate or reasons how this was considered in the analysis. Some interviews were conducted with patients while others were conducted through proxies – no information concerning this assignment was provided. GP response rate was 0%. |
| Luc M, Corriveau H, Boire G et al (2018) [50] | Mixed methods study | U | Y | Y | Y | Y | Y | N | N | Y | Y | Y | N/A | N/A | Moderate | JBI tool for case series used. |
|  |  | Y | Y | Y | U | Y | U | Y | Y | N | Y | N/A | N/A | N/A |  | JBI tool for qualitative studies used. Response rate unclear. |
| Sale JEM, Bogoch E, Hawker G et al (2014) [44] | Qualitative study | Y | Y | Y | Y | Y | N | Y | Y | Y | Y | N/A | N/A | N/A | High |  |
| Vaculik J, Stepan JJ, Dungl P et al (2017) [47] | Quantitative non-randomised study | Y | Y | Y | Y | Y | Y | Y | N | Y | N/A | N/A | N/A | N/A | Low | While the total score (89%) indicates high quality, there is no JBI tool quantitative non-randomised trials and the JBI tool for quasi-experimental studies used. This study had many issues affecting quality; the same GPs could have treated patients in both groups, ~20% of patients had cognitive impairment which would affect the quality of self-reported outcomes (no proxies or attempts to verify reports were made), and response rates were low. Finally, the intervention was limited by contextual factors – GPs are not authorised to prescribe antiresorptives in Czech Republic. It is therefore unsurprising that the intervention aimed at GPs did not increase GP prescribing of these medicines. |
| Zinger G, Sylvetsky N, Levy Y et al (2021) [46] | Quantitative randomised trial | Y | N | Y | N | N | Y | N | Y | Y | Y | Y | Y | Y | High | JBI tool for RCT used. For the purpose of quality assessment this was considered a “reliable” way of measuring the outcomes. |

JBI; Joanna Briggs Institute Global Research Organisation

Y; yes

N; no

U; unclear

N/A; not applicable

1. Overall quality score: low quality (<50%), moderate quality (50-70%), high quality (>70%). This score was then modified based on reviewer assessment and discussion.

**SUPPLEMENT 4**

Reference list of excluded manuscripts.

1. Aubry-Rozier, B., et al., *Impact of a fracture liaison service on patient management after an osteoporotic fracture: the CHUV FLS.* Swiss Med Wkly, 2018. **148**: p. w14579.

2. Ayoub, W., et al., *Improving detection and treatment of osteoporosis: redesigning care using the electronic medical record and shared medical appointments.* Osteoporosis International, 2009. **20**: p. 37-42.

3. Barrack, C.M., et al., *Secondary prevention of osteoporosis post minimal trauma fracture in an Australian regional and rural population.* Australian Journal of Rural Health, 2009. **17**(6): p. 310-315.

4. Beaton, D., et al., *Addition of a fracture risk assessment to a coordinator's role improved treatment rates within 6 months of screening in a fragility fracture screening program.* Osteoporosis International, 2017. **28**(3): p. 863-869.

5. Bellantonio, S., R. Fortinsky, and K. Prestwood, *How well are community‐living women treated for osteoporosis after hip fracture?* Journal of the American Geriatrics Society, 2001. **49**(9): p. 1197-1204.

6. Besser, S.J., J.E. Anderson, and J. Weinman, *How do osteoporosis patients perceive their illness and treatment? Implications for clinical practice.* Archives of Osteoporosis, 2012. **7**(1): p. 115-124.

7. Bliuc, D., et al., *Barriers to effective management of osteoporosis in moderate and minimal trauma fractures: a prospective study.* Osteoporosis International, 2005. **16**(8): p. 977-82.

8. Bullock, L., et al., *Developing a model Fracture Liaison Service consultation with patients, carers and clinicians: a Delphi survey to inform content of the iFraP complex consultation intervention.* Archives of Osteoporosis, 2021. **16**(1): p. 17.

9. Caffetti, C., et al., *Multidisciplinary model for hospital-territory integrated management of patient with bone fragility: primary and secondary prevention of fractures according to severity and complexity.* Reumatismo, 2020. **72**(2): p. 75-85.

10. Chang, C.B., et al., *One-year outcomes of an osteoporosis liaison services program initiated within a healthcare system.* Osteoporosis International, 2021. **32**(11): p. 2163-2172.

11. Chenot, R., et al., *German primary care doctors' awareness of osteoporosis and knowledge of national guidelines.* Experimental and Clinical Endocrinology & Diabetes, 2007. **115**(09): p. 584-589.

12. Cooper, M., A. Palmer, and M. Seibel, *Cost-effectiveness of the Concord Minimal Trauma Fracture Liaison service, a prospective, controlled fracture prevention study.* Osteoporosis international, 2012. **23**: p. 97-107.

13. Crissman, J.K., et al., *Evaluation of the need for a fracture liaison service in a patient-centered medical home.* J Am Pharm Assoc (2003), 2019. **59**(4): p. 565-569.

14. Cuddihy, M.-T., *Barriers to postfracture osteoporosis care in postmenopausal women: Challenges and opportunities.* Journal of General Internal Medicine, 2003. **18**(1): p. 70.

15. Cuddihy, M.-T., et al., *A prospective clinical practice intervention to improve osteoporosis management following distal forearm fracture.* Osteoporosis international, 2004. **15**: p. 695-700.

16. Davidson, E., et al., *Prevention of osteoporotic refractures in regional Australia.* Australian Journal of Rural Health, 2017. **25**(6): p. 362-368.

17. Davis, M.M., et al., *“Did I do as best as the system would let me?” Healthcare professional views on hospital to home care transitions.* Journal of General Internal Medicine, 2012. **27**(12): p. 1649-1656.

18. Dehamchia-Rehailia, N., et al., *Secondary prevention of osteoporotic fractures: evaluation of the Amiens University Hospital's fracture liaison service between January 2010 and December 2011.* Osteoporosis International, 2014. **25**(10): p. 2409-2416.

19. Delbar, A., et al., *Persistence with osteoporosis treatment in patients from the Lille University Hospital Fracture Liaison Service.* Bone, 2021. **144**: p. 9.

20. Drew, S., et al., *Making the case for a fracture liaison service: a qualitative study of the experiences of clinicians and service managers.* Bmc Musculoskeletal Disorders, 2015. **16**: p. 8.

21. Drew, S., et al., *Implementation of secondary fracture prevention services after hip fracture: a qualitative study using extended Normalization Process Theory.* Implementation Science, 2015. **10**: p. 8.

22. Drew, S., et al., *Describing variation in the delivery of secondary fracture prevention after hip fracture: an overview of 11 hospitals within one regional area in England.* Osteoporosis International, 2014. **25**(10): p. 2427-2433.

23. Dunn, P., D. Webb, and T.P. Olenginski, *Geisinger high-risk osteoporosis clinic (HiROC): 2013–2015 FLS performance analysis.* Osteoporosis International, 2018. **29**(2): p. 451-457.

24. Edwards, B.J., et al., *Addressing secondary prevention of osteoporosis in fracture care: follow-up to "own the bone".* J Bone Joint Surg Am, 2011. **93**(15): p. e87.

25. Feldstein, A.C., et al., *Older women with fractures: patients falling through the cracks of guideline-recommended osteoporosis screening and treatment.* JBJS, 2003. **85**(12): p. 2294-2302.

26. Fraser, S. and P.K. Wong, *Secondary fracture prevention needs to happen in the country too: The first two and a half years of the Coffs Fracture Prevention Clinic.* Aust J Rural Health, 2017. **25**(1): p. 28-33.

27. Ganda, K., et al., *Compliance and persistence to oral bisphosphonate therapy following initiation within a secondary fracture prevention program: a randomised controlled trial of specialist vs. non-specialist management.* Osteoporosis International, 2014. **25**(4): p. 1345-1355.

28. Goldshtein, I., et al., *Development and efficacy of a computerized decision support system for osteoporosis management in the community.* Archives of Osteoporosis, 2020. **15**: p. 1-7.

29. Gupta, A., et al., *Digital health interventions for osteoporosis and post-fragility fracture care.* Therapeutic advances in musculoskeletal disease, 2022. **14**: p. 1759720X221083523.

30. Hansen, C.A., et al., *Women’s lived experiences of learning to live with osteoporosis: a longitudinal qualitative study.* BMC Women's Health, 2017. **17**: p. 1-12.

31. Harrington, J.T., et al., *Redesigning the care of fragility fracture patients to improve osteoporosis management: a health care improvement project.* Arthritis Care & Research: Official Journal of the American College of Rheumatology, 2005. **53**(2): p. 198-204.

32. Hjalmarson, H.V., B. Ahgren, and M.S. Kjölsrud, *Developing interprofessional collaboration: a longitudinal case of secondary prevention for patients with osteoporosis.* J Interprof Care, 2013. **27**(2): p. 161-70.

33. Hjalmarson, H.V. and M.S. K, *Forming a learning culture to promote fracture prevention activities.* Health Education (0965-4283), 2012. **112**(5): p. 421-435.

34. Holzmueller, C., et al., *Development of a cloud-based application for the Fracture Liaison Service model of care.* Osteoporosis International, 2016. **27**: p. 683-690.

35. Hooven, F., et al., *Follow-up treatment for osteoporosis after fracture.* Osteoporosis international, 2005. **16**(3): p. 296-301.

36. Hui, N., S. Fraser, and P.K.K. Wong, *Patients discharged from a fracture liaison service still require follow-up and bone health advice.* Arch Osteoporos, 2020. **15**(1): p. 118.

37. Ipsen, J.A., et al., *Rehabilitation for life: the effect on physical function of rehabilitation and care in older adults after hip fracture-study protocol for a cluster-randomised stepped-wedge trial.* Trials, 2022. **23**(1): p. 375.

38. Jaglal, S., et al., *A demonstration project of a multi-component educational intervention to improve integrated post-fracture osteoporosis care in five rural communities in Ontario, Canada.* Osteoporosis International, 2009. **20**: p. 265-274.

39. Jaglal, S.B., et al., *How are family physicians managing osteoporosis? Qualitative study of their experiences and educational needs.* Canadian Family Physician, 2003. **49**(4): p. 462-468.

40. Jaglal, S.B., et al., *Information needs in the management of osteoporosis in family practice: an illustration of the failure of the current guideline implementation process.* Osteoporosis international, 2003. **14**: p. 672-676.

41. Jia, K.Q., et al., *Incorporation of a patient navigator into a secondary fracture prevention program identifies barriers to patient care.* Aging Clin Exp Res, 2020. **32**(12): p. 2557-2564.

42. Lai, P.S.M., S.S. Chua, and S.P. Chan, *Impact of pharmaceutical care on knowledge, quality of life and satisfaction of postmenopausal women with osteoporosis.* International Journal of Clinical Pharmacy, 2013. **35**(4): p. 629-637.

43. LeBlanc, A., et al., *Encounter decision aid vs. clinical decision support or usual care to support patient-centered treatment decisions in osteoporosis: the osteoporosis choice randomized trial II.* PloS one, 2015. **10**(5): p. e0128063.

44. Lee, R., et al., *Geographic scope and accessibility of a centralized, electronic consult program for patients with recent fracture.* Rural and Remote Health, 2016. **16**(1): p. 9.

45. Lih, A., et al., *Targeted intervention reduces refracture rates in patients with incident non-vertebral osteoporotic fractures: a 4-year prospective controlled study.* Osteoporosis international, 2011. **22**: p. 849-858.

46. Lu, K., et al., *A novel fracture liaison service using digital health: impact on mortality in hospitalized elderly osteoporotic fracture patients.* Osteoporosis International, 2023: p. 1-15.

47. Luc, M., et al., *Implementing a fracture follow-up liaison service: perspective of key stakeholders.* Rheumatology International, 2020. **40**(4): p. 607-614.

48. MacIntyre, J., et al., *Optimizing osteoporosis care in a rural primary health care center: Findings of a research study aimed to support seniors.* Nursing Forum, 2019. **54**(4): p. 611-618.

49. Majumdar, S.R., et al., *Multifaceted intervention to improve diagnosis and treatment of osteoporosis in patients with recent wrist fracture: a randomized controlled trial.* Cmaj, 2008. **178**(5): p. 569-575.

50. Majumdar, S.R., et al., *A controlled trial to increase detection and treatment of osteoporosis in older patients with a wrist fracture.* Annals of internal medicine, 2004. **141**(5): p. 366-373.

51. Mauck, K., et al., *The decision to accept treatment for osteoporosis following hip fracture: exploring the woman’s perspective using a stage-of-change model.* Osteoporosis International, 2002. **13**: p. 560-564.

52. Mazor, K.M., et al., *Older women’s views about prescription osteoporosis medication: a cross-sectional, qualitative study.* Drugs & aging, 2010. **27**: p. 999-1008.

53. Meadows, L.M., et al., *The importance of communication in secondary fragility fracture treatment and prevention.* Osteoporosis International, 2007. **18**(2): p. 159-66.

54. Meadows, L.M., et al., *After the fall: women's views of fractures in relation to bone health at midlife.* Women & Health, 2004. **39**(2): p. 47-62.

55. Morell, S., et al., *Adherence to osteoporosis pharmacotherapy one year after osteoporotic fracture - a Swiss trauma center secondary prevention project.* Swiss Med Wkly, 2017. **147**: p. w14451.

56. Naranjo, A., et al., *Best Practice Framework of Fracture Liaison Services in Spain and their coordination with Primary Care.* Archives of Osteoporosis, 2020. **15**(1): p. 7.

57. Naranjo, A., et al., *Results of a model of secondary prevention for osteoporotic fracture coordinated by rheumatology and focused on the nurse and primary care physicians.* Reumatol Clin, 2014. **10**(5): p. 299-303.

58. Narayanasamy, M., et al., *Acceptability and engagement amongst patients on oral and intravenous bisphosphonates for the treatment of osteoporosis in older adults.* Age & Ageing, 2022. **51**(11): p. 1-11.

59. Naunton, M., et al., *Multifaceted educational program increases prescribing of preventive medication for corticosteroid induced osteoporosis.* The Journal of Rheumatology, 2004. **31**(3): p. 550-556.

60. Nielsen, D., et al., *Handling knowledge on osteoporosis–a qualitative study.* Scandinavian Journal of Caring Sciences, 2013. **27**(3): p. 516-524.

61. Otmar, R., et al., *General medical practitioners' knowledge and beliefs about osteoporosis and its investigation and management.* Archives of Osteoporosis, 2012. **7**(1): p. 107-114.

62. Papaioannou, A., et al., *Persistence with denosumab therapy among osteoporotic women in the Canadian patient-support program.* Current medical research and opinion, 2015. **31**(7): p. 1391-1401.

63. Ravn Jakobsen, P., et al., *Development of an mHealth application for women newly diagnosed with osteoporosis without preceding fractures: a participatory design approach.* International journal of environmental research and public health, 2018. **15**(2): p. 330.

64. Ravn Jakobsen, P., et al., *Help at hand: Women’s experiences of using a mobile health application upon diagnosis of asymptomatic osteoporosis.* SAGE open medicine, 2018. **6**: p. 2050312118807617.

65. Reventlow, S. and H. Bang, *Brittle bones: ageing or threat of disease exploring women's cultural models of osteoporosis.* Scandinavian journal of public health, 2006: p. 320-326.

66. Roux, S., et al., *Priming Primary Care Physicians to Treat Osteoporosis After a Fragility Fracture: An Integrated Multidisciplinary Approach.* Journal of Rheumatology, 2013. **40**(5): p. 703-711.

67. Rozental, T.D., et al., *Improving evaluation and treatment for osteoporosis following distal radial fractures. A prospective randomized intervention.* J Bone Joint Surg Am, 2008. **90**(5): p. 953-61.

68. Sale, J., et al., *Patients reject the concept of fragility fracture—a new understanding based on fracture patients’ communication.* Osteoporosis international, 2012. **23**: p. 2829-2834.

69. Sale, J., et al., *Patients do not have a consistent understanding of high risk for future fracture: a qualitative study of patients from a post-fracture secondary prevention program.* Osteoporosis International, 2016. **27**(1): p. 65-73.

70. Sale, J.E., et al., *‘If it was osteoporosis, I would have really hurt myself.’Ambiguity about osteoporosis and osteoporosis care despite a screening programme to educate fragility fracture patients.* Journal of evaluation in clinical practice, 2010. **16**(3): p. 590-596.

71. Sale, J.E.M., et al., *A Postfracture Initiative to Improve Osteoporosis Management in a Community Hospital in Ontario.* Journal of Bone and Joint Surgery (American), 2010. **92**(10): p. 1973-1980.

72. Sale, J.E.M., et al., *Perceived messages about bone health after a fracture are not consistent across healthcare providers.* Rheumatology International, 2015. **35**(1): p. 97-103.

73. Scott, T.A., A. Beveridge, and D. Ní Chrónín, *Suboptimal bone protection in geriatric inpatients and effect of a simple educational and mnemonic strategy.* Australasian Journal on Ageing, 2016. **35**(3): p. E36-E39.

74. Senay, A., et al., *Agreement between physicians’ and nurses’ clinical decisions for the management of the fracture liaison service (4iFLS): the Lucky Bone™ program.* Osteoporosis International, 2016. **27**: p. 1569-1576.

75. Shibli-Rahhal, A., et al., *Testing and treatment for osteoporosis following hip fracture in an integrated US healthcare delivery system.* Osteoporosis international, 2011. **22**: p. 2973-2980.

76. Shu, A.D.-H., et al., *Adherence to osteoporosis medications after patient and physician brief education: post hoc analysis of a randomized controlled trial.* The American journal of managed care, 2009. **15**(7): p. 417.

77. Simonelli, C., et al. *Barriers to osteoporosis identification and treatment among primary care physicians and orthopedic surgeons*. in *Mayo Clinic Proceedings*. 2002. Elsevier.

78. Street, J., et al., *Compliance of an elderly hip fracture population with secondary preventative measures. Efficacy of a simple clinical practice intervention.* Acta Orthop Belg, 2006. **72**(2): p. 204-9.

79. Suzuki, N., et al., *Challenges to prevent secondary fractures in patients with hip fractures in Joetsu Myoko, Japan through the increased use of osteoporosis treatment and collaboration with family doctors.* Journal of Bone and Mineral Metabolism, 2017. **35**(3): p. 315-323.

80. Te Lebanon, O.L., et al., *Virtual Orthopedic-Rehabilitation-Metabolic Collaboration for Treating Osteoporotic HIP Fractures.* Endocrine Practice, 2020. **26(3)**: p. 332-339.

81. Tosi, L.L., et al., *The American orthopaedic association's "Own the Bone" initiative to prevent secondary fractures.* Journal of Bone and Joint Surgery-American Volume, 2008. **90A**(1): p. 163-173.

82. Tulk, C., et al., *Improving osteoporosis management following minimal trauma fracture in a regional setting: The Coffs Fracture Card Project.* Australian Journal of Rural Health, 2013. **21**(6): p. 343-349.

83. van den Berg, P., et al., *A dedicated Fracture Liaison Service telephone program and use of bone turnover markers for evaluating one-year persistence with Oral Bisphosphonates.* Fracture liaison service, 2020: p. 139.

84. Waalen, J., et al., *A Telephone-Based Intervention for Increasing the Use of Osteoporosis Medication: A Randomized Controlled Trial.* American Journal of Managed Care, 2009. **15**(8): p. E60-E70.

85. Werner, P. and I. Vered, *Management of osteoporosis: a survey of Israeli physicians' knowledge and attitudes.* The Israel Medical Association Journal: IMAJ, 2000. **2**(5): p. 361-364.

86. Wilton-Clark, M.S., et al., *Autonomy begets adherence: decisions to start and persist with osteoporosis treatment after group medical consultation.* Archives of Osteoporosis, 2020. **15**(1): p. 10.

87. Wood, H., et al., *Improving community prescribing of post-fracture denosumab after discharge.* British Journal of Hospital Medicine, 2017. **78**(1): p. 20-22.

88. Yadav, L., et al., *Utilising digital health technology to support patient-healthcare provider communication in fragility fracture recovery: systematic review and meta-analysis.* International journal of environmental research and public health, 2019. **16**(20): p. 4047.

**SUPPLEMENT 5**

**Table**: Results of thematic analysis of study outcomes

| **Research question** | **Themes** | **Supporting data** | **Studies** |
| --- | --- | --- | --- |
| The FLS-to-PC transition | Communication issues | *You don’t get a letter, so it’s more the patient turning up going ‘oh, I’ve been contacted for a bone mineral density test because I fractured my whatever’* (GP 5). | [40] |
|  |  | *It takes a while, there is delay, it usually takes a month, and in our age when there is electronic communication it shouldn’t take that long* (GP 3). | [40] |
|  |  | *There seem to be very – two separate camps: there’s what actually happens in trauma and then there’s what happens in primary care, and the communication is difficult (Participant ID: 009).* | [43] |
|  | Siloed or disconnected care | *There seemed to be a lack of, not trust but, there would be concern that continuation of care, or the lines of communication would be, um, maybe interrupted or lost (FLS clinician 6).* | [40] |
|  |  | Patients experienced specialist-led osteoporosis follow-up care as superior to GP-led care with regard to continuity, convenience, and quality of advice. They experienced GP-led osteoporosis care as “messy” as it was associated with an increased number of healthcare transitions, which may be particularly challenging for older persons. | [40] |
|  |  | *There seem to be very – two separate camps: there’s what actually happens in trauma and then there’s what happens in primary care, and the communication is difficult (Participant ID: 009).* | [43] |
|  | GP knowledge gaps | *We took it out of the hands of the GPs because it was being done so poorly ... I don’t think GPs actively try and you know go against what you’ve recommended, but I do think education forms a very, um, fundamental base (FLS clinician 1).* | [40] |
|  |  | *We had a letter saying please do a FRAX risk assessment in something, and I was thinking well OK, I don’t know what a FRAX risk assessment is, but I’ll Google for it and found it, and did it. And then I couldn’t understand why, what I should then do as a result of it* (B008c_GP). | [41] |
|  |  | *And then we never really know whether to stop [bisphosphonates] or not...* (B010c_ GP). | [41] |
|  |  | *Sometimes I might not know what to do with a [diagnostic test] result if it’s a bit difficult ... I’d have to ask advice ... ring up the bone density team, speak to the consultant, just say ‘I’m sorry, I don’t know what to do about this’* (B006c_GP). | [41] |
|  | Limited confidence in primary care follow-up | *Do I trust GPs to implement? I actually trust the GPs more than I trust the patient (FLS clinician 3).* | [40] |
|  |  | *I don’t think there’s an option for it to be initiated in Primary Care for us at the moment because even when we send people home on it the GPs don’t always continue it [Participant ID: 017].* | [43] |
|  |  | *I think then also there’d be concern that the GPs themselves would not be integrating this care into the management plans … (FLS Clinician 6).* | [40] |
|  |  | *Even when we send people home on it the GPs don’t always continue it* (Participant ID: 017). | [43] |
|  |  | Most GPs indicated that they reviewed their patients after fracture presentation to the ED, informed them about their risk of osteoporosis, and considered it their responsibility to investigate and manage this risk with the patient. Baseline patient survey data contradicted this, revealing a low level of patient awareness, and a low rate of investigation and treatment uptake. | [48] |
|  |  | *Well in theory the GPs should be monitoring these patients... But it doesn’t happen. It might happen on the odd GP, but that isn’t happening* (Participant ID: 011). | [43] |
|  | Time and workload pressures | *If you’re working in a practice where your minimum appointment is six minutes, which is generally terrible medicine, which is what you see in some of the clinics in the area (GP 5).* | [40] |
|  |  | *So you’ve got ten minutes to explain to someone that their bones are thinning, they’re at risk of fractures and I’m putting you on this tablet and this is how you have to take it.* (B005c_GP). | [41] |
|  |  | *GPs get probably 400 or 500 letters a day, do they read everything? Hopefully they do (Participant ID: 035).* | [43] |
| Barriers and facilitators to PC follow-up after FLS | Miscommunication and misinformation | Inconsistent information across the healthcare continuum was identified as one of the main barriers to adhering to FLS recommendations. | [50] |
|  |  | *He [primary care physician] said it wasn’t the bones that were bad, it was just sheer bad luck in the way I twisted my foot (ID15).* | [44] |
|  |  | *She [physician] said, typically, a Colles’ fracture isn’t related to osteoporosis” (ID25)* | [44] |
|  |  | *[My GP said] everything was okay [because] I didn’t shrink (ID3).* | [44] |
|  |  | *He [orthopaedic surgeon] told me...that Blacks don’t get osteoporosis... (ID24)* | [44] |
|  |  | *[Her GP] was quite happy with the one part of [her] spine that was slightly osteopenia [but] the rest of the spine was good” (ID15)* | [44] |
|  |  | *[My physician] told me to lose weight...she said it would be easier on my bones and everything else” (ID18).* | [44] |
|  | Understanding roles and responsibilities | *Who’s managing this? Do you want me to manage it or not?... I think that’s part of the issue. I don’t know what the hospital sees its role is ... I don’t have a problem in terms of seeing this as a condition that most of the time I could manage quite happily, so I don’t necessarily see a role for the hospital clinic (GP 4).* | [40] |
|  |  | *I suspect that a lot [of patients] will sort of think, you know, ‘the FLS is looking after this aspect’ and I suspect that that’s part of where things fall off at the end* (FLS clinician 7). | [40] |
|  |  | Patients identified the main prescribers of their osteoporosis treatments as GPs (52%), geriatricians (30%) and ED doctors (13%). | [48] |
|  |  | Among 306 GPs, 259 (85%) considered it their responsibility to investigate and manage fracture risk | [48] |
|  | GP-patient relationship | *He was so rude and so abrasive, that I walked out of there with a really bad attitude and I’ve decided not to do anything about it* (Patient 2). | [40] |
|  |  | *Because I had so much faith in this GP, I decided to do something about it* (Patient 2). | [40] |
|  |  | *I had to go and see another GP... and I realised, yeah, you can have perhaps a more informative relationship with you GP (Patient 7).* | [40] |
|  |  | *My doctor (GP) recommended that I take it. I am obedient. When he asks me to take medication, I take it. I don’t argue with that. (Participant 4, male, 79 years).* | [50] |
|  |  | *My family doctor is no good. Most of the time, I have to see another doctor who prescribes medication for me. I don’t trust her with pills. (Participant 10, female, 60 years).* | [50] |
|  | Patient knowledge and understanding | **Appreciating the link between fracture and osteoporosis** | |
|  |  | *[My GP] said ‘we really have to do something, it’s important’. I mean, I didn’t really think it was all that big of deal but now I know how severe it can become... I thought ‘so what, I’ve got brittle bones’. I didn’t really understand the consequences of falling over and breaking something (Patient 2).* | [40] |
|  |  | Independent predictors for having a primary care physician follow-up were an age older than 50 years (p=0.0003) and a perception of their fracture as osteoporotic (p=0.03). | [32] |
|  |  | Baseline osteoporosis knowledge predicted starting osteoporosis therapy (p = 0.015). | [45] |
|  |  | Patients attributed their fracture to circumstances of the fall: *it was because of freezing rain* and *I think I am very clumsy* | [49] |
|  |  | Reasons provided for not seeing a GP post fracture included … patient belief that they did not need to visit a GP because the fracture had been “fixed” (25%). | [31] |
|  |  | *If I‘d known before, I would have started taking drugs against osteoporosis at that time and I wouldn’t have had a fracture. It would have really helped me. (Participant 12, male, 65 years).* | [50] |
|  |  | *Thirteen (of 16) participants did not identify their fragility fracture as a sign of osteoporosis* | [50] |
|  |  | **Appreciating the seriousness of the condition** | |
|  |  | *Patients tend to, follow-up a little bit more if it hurts or if they actually think it’s important* (GP 1). | [40] |
|  |  | *Obviously as time goes by people are taking medication for something which is an asymptomatic condition and so generally sort of drifting off treatment”* (B023c_Consultant). | [41] |
|  |  | *You think there would be a lot more weight to it if the orthopaedic surgeon says that you need to consider osteoporosis* | [49] |
|  |  | **Understanding follow-up recommendations** | |
|  |  | *What [would I see my GP] for? I speak to [FLS clinician] when I’ve seen them... I mean there’s no reason for me to go and see the GP (Patient 3).* | [40] |
|  |  | Forty-five (14.2%) patients did not visit their GP, either because they already had anti-osteoporotic medication prior to this fracture event (32), refused (10) or did not understand they were supposed to go to their GP for the prescription and further support (3)*.* | [33] |
|  |  | *(rhetorically) What can he [GP] do at this point?* | [49] |
|  |  | Not understanding why the medication was needed was a reason cited for treatment cessation. | [31] |
|  | Healthcare policies and funding | *As far as I know osteoporosis isn’t a QOF [additional payment targeted to the treatment of certain conditions] thing so there’s no incentive there to follow up* (B010c_GP) | [41] |
|  | GP engagement | **Willingness to manage osteoporosis** | |
|  |  | Most GPs indicated they would use simple guidelines, if available. | [48] |
|  |  | *A few GPs, er, have reluctance for them to have someone else manage their client’s osteoporosis or be guided by that. I feel like they want to manage it themselves* (FLS clinician 4). | [40] |
|  |  | GPs reported high levels of confidence managing osteoporosis and were comfortable prescribing most medications and monitoring long-term post-fracture care. | [40] |
|  |  | **Prioritising osteoporosis** | |
|  |  | *Usually you just go tick, tick, tick, and see the next patient, but if you’re thorough you might put in a diagnosis and a recall, otherwise it might wait until the patient turns up (GP 7).* | [40] |
|  |  | *I think, there’s not a lot of general publicity about it, you know, everyone’s focussed on cancer, and that, you know, which is obviously important, but I think people significantly underestimate the associated morbidity with osteoporosis (GP 4).* | [40] |
|  |  | *There definitely has to be a trigger to remind people to think about osteoporosis amongst the 209 other things (GP).* | [49] |
|  |  | **Available resources** |  |
|  |  | *Well if we had infinite resources then I would have a sort of annual review [of patients] in general practice but that’s never going to happen because GPs are just too busy at the moment* (B006c_GP). | [41] |
|  |  | *I just don’t have the resource to [follow-up] (Participant ID: 023).* | [43] |
|  | Accessing primary care from or residential facilities | Reasons provided for not seeing a GP post fracture included: patient being discharged to a rehabilitation centre (35%) and patient was a resident of a nursing home (15%). | [31] |
|  | GP gender | Having a female physician predicted BMD testing (p = 0.032). | [45] |
| Interventions to enhance integration | Blood marker monitoring | *People like to know that someone’s checking on them and they’re getting a bit of feedback and if they’re going to be asked to take the medication that somebody is checking that it’s working, so they’re not very burdensome to have a blood test and a phone call, definitely less burdensome than coming and having a bone density scan (B023c_ Consultant).* | [41] |
|  | Clinical protocol for low-trauma fractures | No significant differences between groups in relation to recommendations for or initiation of osteoporosis investigations. There was no significant difference between groups in to the proportion who visited a GP for follow-up. | [31] |
|  | Detailed individualised (cf general) management recommendations for GPs | Compared with general recommendations, providing patients and their GPs with detailed individualised recommendations for osteoporosis investigation and management following fracture did not lead to an increase in use of BMD testing, x-ray imaging, specialist care, or prescription for anti-osteoporosis medication. | [47] |
|  | Telephone coaching | Significantly more patients in group 1 (Type B FLS with monthly telephone coaching) were receiving recommended treatment at 4 months, compared with those in group 2 (Type C FLS) (77.1% vs 6.2%, p <0.0001). | [46] |
